# A Longitudinal Trial Comparing Chloroquine as Monotherapy or in Combination with Artesunate, Azithromycin or Atovaquone-Proguanil to Treat Malaria

**DOI:** 10.1371/journal.pone.0042284

**Published:** 2012-08-17

**Authors:** Miriam K. Laufer, Phillip C. Thesing, Fraction K. Dzinjalamala, Osward M. Nyirenda, Rhoda Masonga, Matthew B. Laurens, Abbie Stokes-Riner, Terrie E. Taylor, Christopher V. Plowe

**Affiliations:** 1 Malaria Group, Howard Hughes Medical Institute/Center for Vaccine Development, University of Maryland School of Medicine, Baltimore, Maryland, United States of America; 2 Blantyre Malaria Project, University of Malawi College of Medicine, Blantyre, Malawi; 3 EMMES Corporation, Rockville, Maryland, United States of America; 4 College of Osteopathic Medicine, Michigan State University, East Lansing, Michigan, United States of America; University of California Los Angeles, United States of America

## Abstract

**Background:**

The predominance of chloroquine-susceptible falciparum malaria in Malawi more than a decade after chloroquine's withdrawal permits contemplation of re-introducing chloroquine for targeted uses. We aimed to compare the ability of different partner drugs to preserve chloroquine efficacy and prevent the re-emergence of resistance.

**Methodology/Principal Findings:**

Children with uncomplicated malaria were enrolled at a government health center in Blantyre, Malawi. Participants were randomized to receive chloroquine alone or combined with artesunate, azithromycin or atovaquone-proguanil for all episodes of uncomplicated malaria for one year. The primary outcome was incidence of clinical malaria. Secondary endpoints included treatment efficacy, and incidence of the chloroquine resistance marker *pfcrt* T76 and of anemia. Of the 640 children enrolled, 628 were included in the intention-to-treat analysis. Malaria incidence (95% confidence interval) was 0.59 (.46–.74), .61 (.49–.76), .63 (.50–.79) and .68 (.54–.86) episodes/person-year for group randomized to receive chloroquine alone or in combination with artesunate, azithromycin or atovaquone-proguanil respectively and the differences were not statistically significant. Treatment efficacy for first episodes was 100% for chloroquine monotherapy and 97.9% for subsequent episodes of malaria. Similar results were seen in each of the chloroquine combination groups. The incidence of *pfcrt* T76 in pure form was 0%; mixed infections with both K76 and T76 were found in two out of 911 infections. Young children treated with chloroquine-azithromycin had higher hemoglobin concentrations at the study's end than did those in the chloroquine monotherapy group.

**Conclusion/Significance:**

Sustained chloroquine efficacy with repeated treatment supports the eventual re-introduction of chloroquine combinations for targeted uses such as intermittent preventive treatment.

**Trial Registration:**

ClinicalTrials.gov NCT00379821

## Introduction

In the long struggle to control and ultimately eliminate malaria, the development of resistance to antimalarial drugs has caused repeated setbacks. Chloroquine and sulfadoxine-pyrimethamine (SP) have been retired from use as first-line treatment for malaria in most areas due to widespread resistance. There is evidence that reduced susceptibility to the leading class of antimalarial drugs that replaced chloroquine and SP, the artemisinins, has emerged in Southeast Asia [1], [2]. Malawi was the first African country to stop using chloroquine due to high rates of drug resistance and, 12 years after chloroquine use was discontinued, chloroquine-susceptible parasites now predominates there [3], [4]. A review of chloroquine use and patterns of chloroquine drug resistance in sub-Saharan Africa indicate that if the current trends in decreased chloroquine use continue alongside the increased availability and use of artemisinin-based combination therapies, chloroquine-susceptible malaria may regain prominence in Africa [5].

If chloroquine is reintroduced, whether for routine treatment or for targeted prevention, it will be prudent to administer it with a partner drug to protect against the re-emergence of resistance. Combination therapy is recommended to effectively treat individual infections and to prevent the emergence and spread of resistance [6]. Evidence-based strategies to select the optimal combinations to deter the development and spread of parasites resistant to the individual drugs have not been developed. To date, countries have adopted specific combinations on the basis of drug availability and data from short-term efficacy studies. Current artemisinin-based combination therapies combine short-acting, highly effective artemisinins with longer-acting drugs that offer post-treatment prophylactic benefit, protecting against late recrudescent infections as well as new infections for up to several weeks following treatment. In areas of high malaria transmission, it may be important to combine medications with matching pharmacodynamic characteristics to avoid exposing longer-acting partner drugs to an increased risk of developing resistance [7].

To gain insight into the pharmacokinetic and pharmacodynamic properties that might protect against the re-emergence of chloroquine resistance, we selected three partner drugs to use in combination with chloroquine. Artesunate is a highly effective, very short-acting medication that rapidly decreases parasite biomass, but has a duration of action of only a few hours [8]–[10]. Azithromycin is slow-acting, moderately efficacious in its antimalarial activity, and present at therapeutic concentrations in the blood for approximately five to ten days based on data from bacterial infection [11]. Finally, atovaquone, co-formulated with proguanil (Malarone), has a long terminal elimination phase and has antimalarial activity in the blood lasting for weeks that approximates that of chloroquine [12]–[14].

Malawi provided a unique opportunity to evaluate the effect of drug combinations on the evolution of drug resistance. In this small southern African country, *P. falciparum* parasites are predominantly susceptible to chloroquine, a drug which, unlike the artemisinins, has established molecular markers of drug resistance [15]. Chloroquine-resistant parasites that may emerge after repeated treatment with different combination therapies can be detected, even if clinical treatment failure does not occur.

This study was designed as a longitudinal trial, rather than as the evaluation of efficacy of a treatment regimen against a single episode of malaria. For pivotal studies that examine the application of new drugs and treatment strategies, we have advocated for the use of longitudinal studies [16]. In addition to measuring efficacy of individual treatments, longitudinal studies measure sustained efficacy with repeated use of the same regimen over time, a scenario that reflects the ideal pattern of use of antimalarial medication in highly endemic areas. The primary outcome of interest, the annual incidence of malaria episodes, and the secondary outcomes of anemia and severe malaria, are all highly relevant to public health policy-makers as they reflect not only the burden of disease but also the demand on health resources.

## Methods

### Participants

Ndirande is a peri-urban hillside township of Blantyre, the second largest city in Malawi. Malaria transmission is year-round, with a seasonal peak from December to March corresponding to the rainy season. The Ndirande Health Center is the sole government health facility serving the township's estimated population of 200,000. Children attending the pediatric clinic who were suspected of having malaria were offered screening and enrollment in this study.

The protocol for this trial and supporting CONSORT checklist are available as supporting information: see Checklist S1 and Protocol S1. Children aged ≥6 months through 5 years with signs or symptoms consistent with malaria were eligible for enrollment (axillary temperature ≥37.5°C by digital thermometer, report of fever within the last two days, or other constitutional symptoms). Further eligibility criteria included: weight ≥5 kg; positive malaria smear for *P. falciparum* mono-infection with parasite density 2,000–200,000/mm^3^; planning to remain in the study area for one year; willingness to return for four-weekly routine visits and unscheduled sick visits; and parental/guardian consent. Exclusion criteria included danger signs of severe malaria (hemoglobin ≤5 g/dL, prostration, respiratory distress, bleeding, seizures or coma, inability to eat or drink or persistent vomiting); known allergy to a study drug; on chronic medication with a drug that has antimalarial activity; abnormal liver enzymes or renal function; and evidence of severe malnutrition or chronic disease. Children with known HIV infection were not enrolled because trimethoprim-sulfamethoxazole, an antimicrobial with antimalarial activity, is recommended for all HIV-infected children in Malawi.

### Ethics

All parents or guardians provided written informed consent prior to the initiation of any study-related procedures. The study protocol was reviewed and approved by the University of Malawi College of Medicine Research and Ethics Committee and the Institutional Review Board of the University of Maryland, Baltimore. The study was registered on ClinicalTrials.gov (NCT00379821).

### Intervention

This study was a randomized longitudinal open-label drug efficacy trial among children who presented to the Ndirande Health Center with uncomplicated falciparum malaria. Participants were randomized with equal allocation to the four drug treatment arms: chloroquine monotherapy (Nivaquine™, Sanofi-Aventis 100 mg tablet:10 mg/kg on days 0 and 1; 5 mg/kg on day 2), chloroquine plus artesunate (Arsumax™, Sanofi Aventis or artesunate, Guillin Pharmaceuticals, 50 mg tablets: 4 mg/kg/day for 3 days), chloroquine plus azithromycin (Zithromax™, Pfizer, 200 mg/5 cc suspension: 30 mg/kg/day for 3 days), and chloroquine plus atovaquone-proguanil (Malarone™, GSK, pediatric tablets 62.55 mg/25 mg and full strength 250 mg/100 mg dosing in accordance with the package insert: 15–25 mg/kg atovaquone and 5–10 mg/kg proguanil for three days). Doses were rounded to the nearest one quarter tablet. Treatment was not blinded because of the use of tablets and liquids in different arms as well as the frequent need to crush tablets (to administer to young children) precluded over-encapsulation. At each malaria episode during the 12-month study period, participants received the same treatment as the one assigned at enrollment. Study medications were administered under direct observation. For all episodes of malaria, drug efficacy was assessed for 28 days. After the participant was enrolled in the study, an episode of malaria that occurred more than 14 days after a diagnosis of malaria was considered a subsequent episode and treated with the assigned treatment regimen. Participants returned to the clinic every four weeks for active follow-up and were encouraged to come to the clinic whenever they were ill. At every visit, the parents were questioned about medical care received outside the study and the health records were reviewed. If a participant was treated for malaria outside the study, the episode was recorded as a malaria illness and included in the intention-to-treat analysis. Participants who missed two consecutive 4-weekly routine visits were discontinued from the study.

### Safety laboratory monitoring

Full blood count with automated thre-part differential, ALT and creatinine were obtained at day 0 and day 14 of every clinical malaria episode and also at study weeks 16, 32 and 52. Normal values for hematological and biochemical evaluations were based on data collected from healthy children in rural Mozambique [17] except that platelet and neutrophil count values were based on pediatric toxicity tables published in 2003 by the National Institutes of Health. Basic neurological assessments were done at day 28 of each illness and at study weeks 16, 32 and 52.

### Malaria outcomes assessment

At enrollment and during subsequent episodes of uncomplicated malaria (defined as malaria illness in the absence of danger signs), diagnosis and treatment initiation occurred on day 0 and all doses of medication were administered under direct observation. Participants were followed actively on days 1, 2, 3, 7, 14, 21 and 28 at the study clinic, and by daily availability of a study clinician to evaluate and treat as needed. Malaria smears, dried blood spots on filter paper (Whatman 3MM) and hemoglobin measurement (HemoCue, Angelholm, Sweden) were obtained at every follow up visit except day 1. The 2004 World Health Organization definitions of treatment outcomes were used [18].

### Genotyping

All specimens from day 0 of each episode of malaria illness underwent pyrosequencing to determine the allele at position 76 of the *P. falciparum* chloroquine resistance transporter gene (*pfcrt*); *pfcrt* T76 is the primary marker for chloroquine resistance [15]. This method is able to detect a minority allele when it is present in at least 20% of the infection. All day 0 specimens were included, even if the treatment outcomes were not evaluable. Specimens from infections that included non-falciparum species were included in this analysis. Recurrent infections were genotyped to distinguish recrudescence from reinfection using merozoite surface protein 2 (*msp*2), as described previously [19]. Fragment sizes were analyzed using Qiaxcel. If, within a range of ten base pairs, the same alleles were present at the initial and recurrent episodes of infection the infection was considered recrudescent. Protocols for all molecular methods are available on our website (http://medschool.umaryland.edu/malaria/protocols.asp).

### Objectives

The primary objective of the study was to compare the annual incidence of malaria clinical episodes between the CQ monotherapy arm and each of the three combined treatment arms. We hypothesized that the annual incidence rates of malaria episodes will differ between the group treated with chloroquine monotherapy and the group treated with chloroquine combined with a partner study drug.

The secondary objectives were to assess the anti-malarial drug efficacy at first and subsequent administrations by treatment arm, to measure the effect of each treatment arm on anemia and the prevalence of chloroquine resistance and to assess the safety of repeated use of the drugs in each study arm.

### Sample size

We estimated a mean rate of 1.5 malaria episodes per year with chloroquine alone. We derived the sample size required to detect a decrease in the mean to 1.0 episodes per year assuming a Poisson distribution and using a 2 sided test with alpha = 0.05/3 = .0166. The test size was adjusted to accommodate the 3 treatment versus control comparisons. With the above requirements and for a 2 treatment contrast, 120 subjects with complete observation were required per treatment arm to have 90% power. To account for migration and other causes of attrition, we enrolled an additional 33% (40 subjects) in each regimen for a total of 160 per arm, and an overall sample size of 640 subjects.

### Randomization and blinding

The randomization sequence was generated by the trial statistician in SAS using blocked randomization; assignments were concealed using a pull-tab treatment list until participants were enrolled. This was an open-label trial. The laboratory technicians who read the malaria smears were blinded to study drug allocation.

### Statistical analysis

The primary endpoint for the study was the incidence of clinical malaria episodes per year of follow-up. A Poisson regression model offset by follow-up time was used to estimate the annual incidence of malaria in each treatment arm and to compare each combination arm to the chloroquine monotherapy arm. Results were reported using both the intention-to-treat and per-protocol populations. Intention-to-treat analysis used all evaluable subjects. All malaria episodes subsequent to initial treatment, including non-*P. falciparum* infections and episodes diagnosed outside of the study clinic, were included. Per-protocol analysis excluded subjects who did not receive the full course of treatment and included only those malaria episodes subsequent to initial treatment caused by *P. falciparum* mono-infection as diagnosed at the study clinic. Follow-up time was from enrollment to date of last contact, or until receipt of off-protocol anti-malarial medication. Time at risk excluded time under treatment for malaria.

Kaplan-Meier estimates were used to calculate the probability of malaria-free survival by treatment arm. Cox proportional hazards were used to estimate the risk of malaria after initial treatment in each treatment arm and the incidence of severe malaria.

Rates of adverse events and serious adverse events were compared by the Kruskal-Wallis test. For hemoglobin concentration and other laboratory tests conducted for safety, a generalized estimating equations (GEE) model with Gaussian link and exchangeable correlation structure was fit to estimate the effect of repeated use of study drugs on the mean clinical laboratory value, adjusted for treatment arm and age. Data were analyzed in SAS version 9.2 (SAS Institute, Carey, NC) and R version 2.10.0.

## Results

### Participant flow and recruitment

**Figure 1:**
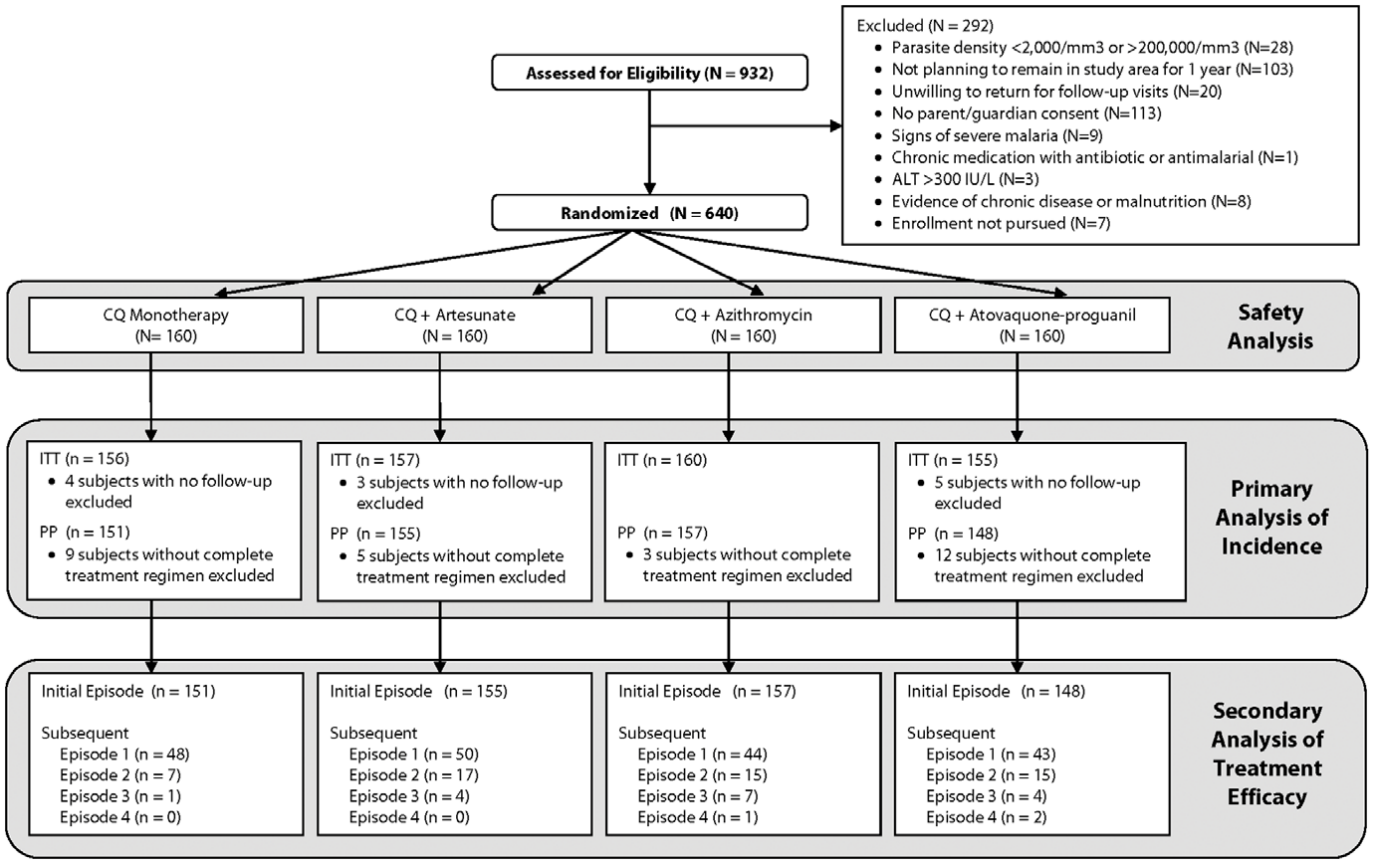
Consort Diagram.

Nine hundred thirty-two potential participants underwent screening and 640 were enrolled between February 2007 and August 2008; follow-up was completed in 2009 (figure 1, CONSORT diagram). The most common reasons for failing to enroll were not planning to remain in the study area for one year (n = 103), and parent/guardian unwilling to provide consent because of concern the other parent/guardian would object (n = 96).

### Baseline data

Six hundred forty children were successfully enrolled in the study. The age range was 0.5–5.3 years, the mean 2.7 years. The enrollment demographic data and malaria characteristics at enrollment were similar in each treatment arm (table 1).

**Table 1:** Demographics and Clinical Characteristics of Initial Malaria Episode.

		*Treatment Group*				
		*CQ Monotherapy* *(N = 160)*	*CQ+Artesunate* *(N = 160)*	*CQ+Azithromycin* *(N = 160)*	*CQ+Atovaquone-proguanil* *(N = 160)*	*All Subjects* *(N = 640)*
**Gender**						
All Subjects	Female	77 (48.1%)	70 (43.8%)	73 (45.6%)	82 (51.3%)	302 (47.2%)
N(%)	Male	83 (51.9%)	90 (56.3%)	87 (54.4%)	78 (48.8%)	338 (52.8%)
**Age**						
All Subjects	>6 to 12 months	16 (10.0%)	14 (8.8%)	12 (7.5%)	14 (8.8%)	56 (8.8%)
N(%)	>1 to 3 years	79 (49.4%)	81 (50.6%)	79 (49.4%)	78 (48.8%)	317 (49.5%)
	>3 to 5 years	65 (40.6%)	65 (40.6%)	69 (43.1%)	68 (42.5%)	267 (41.7%)
**Weight (kg)** *						
≥6 to 12 months	Mean (SD)	8.0 (0.67)	8.4 (1.23)	8.3 (0.94)	8.4 (1.80)	8.2 (1.21)
≥1 to 3 years	Mean (SD)	11.1 (1.75)	11.0 (1.65)	11.3 (1.47)	10.9 (1.47)	11.1 (1.59)
≥3 to 5 years	Mean (SD)	13.4 (1.60)	13.5 (1.57)	13.3 (1.62)	13.6 (1.50)	13.4 (1.57)
**Stature (cm)** *						
≥6 to 12 months	Mean (SD)	69.7 (3.45)	69.5 (4.03)	69.5 (2.85)	70.8 (8.53)	69.9 (5.12)
≥1 to 3 years	Mean (SD)	83.0 (5.95)	82.0 (5.94)	83.6 (5.31)	81.7 (5.16)	82.6 (5.63)
≥3 to 5 years	Mean (SD)	95.1 (7.21)	94.2 (5.19)	94.3 (6.24)	95.3 (5.29)	94.7 (6.02)
**Parasite Density/mm^3^**						
All subjects	Geometric Mean	21,413	21,470	23,399	19,341	21,358
	(min, max)	(1,880, 191,800)	(2,006, 191,800)	(2,401, 191,500)	(2,040, 189,198)	(1,880, 91,800)
**Hemoglobin (g/dL)**						
≥6 to 12 months	Mean (SD)	9.0 (1.27)	8.7 (1.95)	9.2 (1.29)	8.9 (1.48)	9.0 (1.49)
	(min, max)	(6.2,10.5)	(5.5, 10.9)	(7.2, 11.1)	(7.0, 11.4)	(5.5, 11.4)
≥1 to 3 years	Mean (SD)	9.8 (1.60)	9.6 (1.88)	10.0 (1.78)	9.7 (1.87)	9.7 (1.78)
	(min, max)	(5.5, 14.3)	(5.5, 16.0)	(5.6, 14.3)	(5.6, 15.0)	(5.5, 16.0)
≥3 to 5 years	Mean (SD)	9.5 (1.70)	10.5 (1.75)	10.1 (2.03)	10.0 (1.71)	10.0 (1.83)
	(min, max)	(5.7, 13.9)	(5.7, 14.2)	(5.8, 16.5)	(6.3, 13.0)	(5.7, 16.5)
**Enrollment Season** ††						
All subjects	Peak Malaria Season	52 (32.5%)	52 (32.5%)	54 (33.8%)	51 (31.9%)	209 (32.7%)
	Off-peak Malaria Season	108 (67.5%)	108 (67.5%)	106 (66.3%)	109 (68.1%)	431 (67.3%)

* 7 subjects missing baseline weight and stature measurements.

†† Peak malaria season is defined as 01 Dec through 31 March.

### Numbers analyzed

Six hundred twenty eight subjects who returned for at least one visit after day 0 and were included in the intention to treat (ITT) analysis. Six hundred eleven evaluable subjects with a malaria episode caused by *P. falciparum* received all three doses of study treatment and were included in the per-protocol (PP) cohort for initial malaria illness episode. The PP population for subsequent episodes is shown in Figure 1.

### Efficacy endpoints

There were no statistically significant differences in the incidence of malaria between the chloroquine monotherapy arm and any of the combination therapy arms in the ITT or PP populations (table 2). Treatment outcomes for first and subsequent episodes of malaria are summarized in table 3, including the PCR-adjusted results. All four treatment arms showed high efficacy for the treatment of the initial infection. There were 258 subsequent episodes of malaria in during the one year follow up period and the assigned treatment arms continued to have high efficacy with repeated use. There were too few treatment failures to conduct a statistically meaningful comparison of treatment outcomes.

**Table 2:** Malaria Incidence per Year of Follow-up.

	*CQ Monotherapy*	*CQ+Artesunate*	*CQ+Azithromycin*	*CQ+Atovaquone-Proguanil*
**Intention-to-treat** *				
No. subjects	156	157	160	155
No. episodes	67	77	77	73
Subject-years of follow-up	113.7	125.5	122.1	106.6
Incidence	0.59	0.61	0.63	0.68
(95% CI)	(0.46,0.75)	(0.49,0.77)	(0.50,0.79)	(0.54,0.86)
Relative risk	–	1.04	1.07	1.16
(95% CI)		(0.75,1.45)	(0.77,1.49)	(0.83,1.62)
**Per-Protocol** †				
No. subjects	151	155	157	148
No. episodes	41	57	52	51
Subject-years of follow-up	85	101.9	99.3	84.6
Incidence (95% CI)	0.48 (0.36,0.65)	0.56 (0.43,0.72)	0.52 (0.40,0.68)	0.60 (0.46,0.79)
Relative risk (95% CI)	–	1.16 (0.78,1.71)	1.09 (0.73,1.62)	1.25 (0.84,1.86)

Incidence and relative risk estimated from a Poisson regression model with follow-up time as offset term.

* Intention-to-treat analysis uses all evaluable subjects. All malaria episodes subsequent to initial treatment, including non-P. *falciparum* infections and episodes diagnosed outside of the study clinic, are included, and follow-up time is days from enrollment to date of last contact.

† Per-protocol analysis excludes subjects who did not receive full course of treatment, and includes only malaria episodes subsequent to initial treatment caused by P. *falciparum* mono-infection and diagnosed at the study clinic. Follow-up time is days from enrollment to date of last contact, or receipt of off-study anti-malarial medication.

**Table 3:** Summary of 28-Day Treatment Outcomes*.

	*CQ Monotherapy*	*CQ+Artesunate*	*CQ+Azithromycin*	*CQ+Atovaquone-Proguanil*
*n (%)*				
**Initial Episode**				
Completed treatment	151	155	157	148
Known 28-day treatment outcome	135 (89.4)	144 (92.9)	138 (87.9)	133 (89.9)
Adequate clinical and parasitological response	135 (100.0)	143 (99.3)	137 (99.3)	133 (100.0)
Early Clinical Failure	0	0	0	0
Late Clinical Failure	0	1 (0.7)	1 (0.7)	0
PCR classification		Recrudescent	New	
**First subsequent episode**				
Completed treatment	48	50	44	43
Known 28-day treatment outcome	41 (85.4)	46 (92.0)	39 (88.6)	42 (97.7)
Adequate clinical and parasitological response	39 (95.1)	46 (100.0)	37 (94.9)	42 (100.0)
Early Clinical Failure	1 (2.4)	0	0	0
Late Clinical Failure	1 (2.4)	0	2 (5.1)	0
PCR classification	New		1 New, 1 Recrudescent	
**Second subsequent episode**				
Completed treatment	7	17	15	15
Known 28-day treatment outcome	6 (85.7)	17 (100.0)	12 (80.0)	14 (93.3)
Adequate clinical and parasitological response	6 (100.0)	17 (100.0)	12 (100.0)	14 (100.0)
**Third subsequent episode**				
Completed treatment	1	4	7	4
Known 28-day treatment outcome	1 (100.0)	4 (100.0)	7 (100.0)	4 (100.0)
Adequate clinical and parasitological response	0	4 (100.0)	6 (85.7)	4 (100.0)
Late Clinical Failure	1 (100.0)	0	1(14.3)	0
PCR classification	Recrudescent		Recrudescent	
**Fourth subsequent episode**				
Completed treatment	0	0	1	2
Known 28-day treatment outcome	-	-	1 (100.0)	2 (100.0)
Adequate clinical and parasitological response	-	-	1 (100.0)	2 (100.0)

* WHO 2004 treatment outcome definitions. No subjects experienced late parasitological failure without fever at any episode.

**Figure 2:**
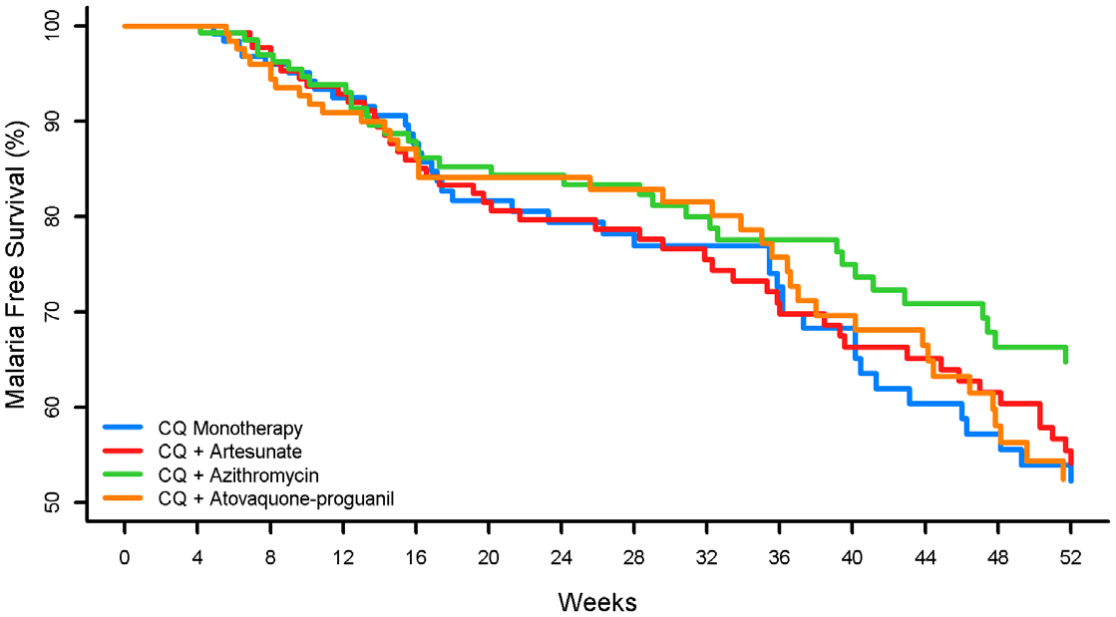
Malaria-free survival from initial episode to first subsequent episode. CQ, Chloroquine.

The time from initial to first subsequent malaria episode was the same for all arms (figure 2). Following treatment for the second episode, the risk of developing a third episode was higher in the chloroquine-azithromycin arm compared with the chloroquine monotherapy group (hazard ratio 2.5, 95% CI 1.0–6.2, p = 0.04), but the difference was not significant because the lower bound included one and the p-value was above our a priori cut-off of 0.0166.

**Figure 3:**
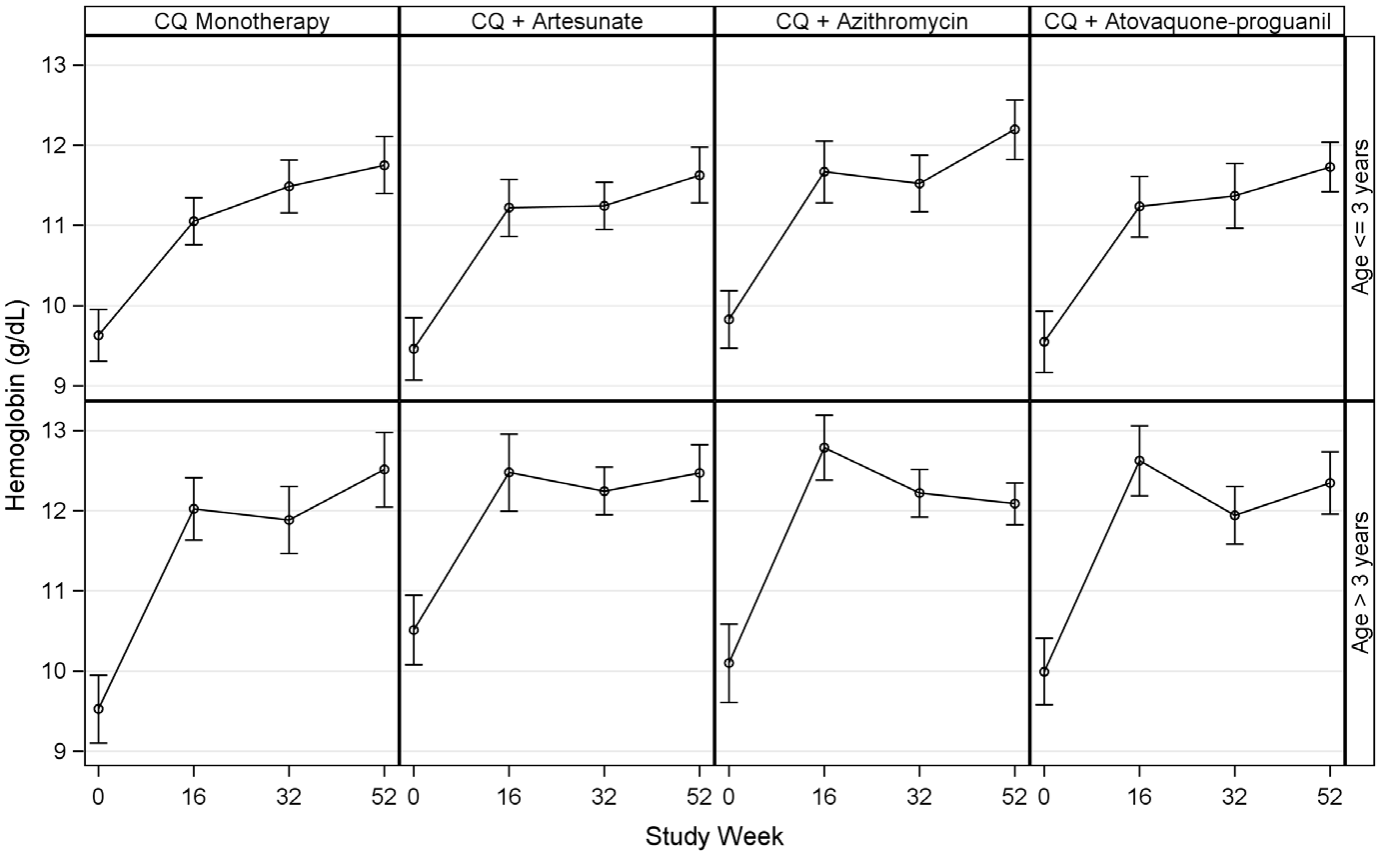
Mean and 95% confidence interval for hemoglobin measured at baseline and routine study follow-up visits.

Among all age groups, there was no difference in the mean concentration in the mean hemoglobin at the end of the study by treatment arm (p = 0.60 for GEE). Hemoglobin concentration at the end of the study period was higher among children ≤3 years of age in the chloroquine-azithromycin arm (mean 12.2 g/dL, 95%CI 11.8–12.6) compared to monotherapy arm (mean 11.8 g/dL, 95%CI 11.7–12.0, p = 0.04 for GEE) (figure 3). There were no differences in the other age groups or treatment arms or when comparing rates of anemia, using moderate and mild anemia cut-offs (data not shown).

### Chloroquine resistance genotyping

Nine hundred twenty-four episodes of malaria, including seven episodes of late treatment failure, occurred. DNA extracted from 911 filter paper specimens was amplified successfully to evaluate the allele at codon 76 of *pfcrt*. Among the evaluated samples, the chloroquine-susceptible form (*pfcrt* K76) was present as the only detectable genotype in 909 (99.8%); the remaining two were mixed infections (i.e., included both K and T). No infections contained the chloroquine-resistant genotype exclusively. One mixed infection occurred 28 days after an initial treatment with chloroquine-artesunate and was classified as a new infection based on genotyping. The participant was subsequently treated with chloroquine-artesunate and had an adequate clinical and parasitological response at 28 days and no further episodes of malaria during the study. The second mixed infection occurred in a participant in the chloroquine-azithromycin arm. The treatment was the third the participant had received: the first occurred at enrollment, the second occurred three months after enrollment, and the episode with the mixed *pfcrt* genotype occurred 3.5 months later. The participant had an adequate clinical and parasitological response and had no further malaria episodes during the study.

### Adverse Events

There were 49 serious adverse events (SAEs) reported in 46 subjects. This included eight evaluable cases of severe malaria in the study, five in the chloroquine monotherapy arm, one in the chloroquine-azithromycin group and two in the chloroquine-atovaquone-proguanil group. The difference between the treatment groups was not significant given the low number of events; however, there were no cases of severe malaria in the chloroquine-artesunate arm. Five SAEs were considered to be possibly associated with study product (vomiting [4]; anemia [1]). Three deaths occurred, two in the monotherapy arm (sequelae of recently diagnosed HIV infection; brain tumor) and one in the chloroquine-azithromycin arm (unknown etiology, occurred outside the study area).

Minor differences between treatment groups were seen in drug-related adverse events. The rate of treatment-related pruritis was lowest in the chloroquine-artesunate group, with 2.5% of participants experiencing the event compared to a range of 12.5–16.3% (p = 0.0005). Neutropenia, possibly related to the study drug based on temporal proximity, occurred in seven participants in the azithromycin arm; all resolved. Although neutropenia occurred infrequently in other treatment groups, none of those episodes was suspected of being associated with the study treatment. No difference in the results of the neurological assessments was detected between the groups. In the GEE models of the safety laboratory results, the effect of episode and the interaction of episode with treatment were not significant in any of the models, suggesting that repeated use of any study treatment did not affect the results.

## Discussion

### Interpretation

In the controlled setting of this clinical trial, the annual incidence of malaria was the same in all treatment arms, including chloroquine alone. Single episode treatment efficacy of chloroquine, both at the initial infection and among the participants who had subsequent episodes of malaria was high and sustained when it was administered repeatedly either alone or as part of combination therapy over twelve months in Blantyre, Malawi. These results further confirm the molecular and clinical evidence demonstrating the complete predominance of chloroquine-susceptible malaria in Malawi for the last ten years, and suggest that chloroquine could have a future role for malaria treatment and prevention in Africa. In this longitudinal study, where parasites were susceptible and in which effective drugs were administered appropriately, chloroquine-resistant parasites did not re-emerge despite repeated treatment in approximately half of the study participants over the course of a year. Because clinical treatment failure may be a relatively insensitive tool for the detection of drug resistance, especially in a population with some pre-existing immunity, we also tested for the molecular marker of chloroquine resistance. Our molecular assay was sensitive enough to detect a minority genotype representing at least 20% of a mixed infection. However, we found only two mixed infections containing chloroquine-resistant parasites among more than 900 episodes of malaria, and no examples of predominantly or exclusively resistant infections despite repeated use of a drug with a half-life of one to two months. This is evidence that resistant parasites are almost entirely absent in this human population.

Because the annual incidence of malaria was lower than we had expected, it is possible that the study did not have sufficient power to detect differences in either the annual incidence of infection or treatment efficacy during subsequent episodes. However, using the observed rate of malaria in both the ITT and PP populations, we should have been able to detect meaningful difference between the chloroquine monotherapy group and any of the combination groups, if they existed. Based on an annual incidence of malaria of 0.6 in the chloroquine monotherapy group, we had 88% power to detect a difference of 0.2 episodes per person year between the two groups, with a type 1 error rate of 0.0166. For the PP analysis, we had 90% power to detect a change from 0.5 to 0.3 episodes per year. The number of treatments required to permit the re-emergence has not been estimated and as a result the study may have not had power to produce sufficient selective pressure. However, we did not have data to make these assumptions *a priori*.

Because clinical efficacy and susceptibility were the same in all treatment arms, we had the opportunity to examine additional secondary benefits and the safety of the various combinations, independent of their antimalarial effect. In the chloroquine-artesunate group, there were no episodes of severe malaria. Severe malaria was uncommon in this clinical trial, probably because illnesses were diagnosed and treated promptly, and given their rarity, the study did not have adequate power to detect differences between groups. Nevertheless, these findings suggest that the addition of artesunate, the most rapid-acting of all the drugs included in this study, may further protect against severe disease, even when case detection and treatment are optimal.

Anemia is another important morbidity associated with repeated malaria infections. We found a significantly higher hemoglobin concentration in children aged ≤3years in the chloroquine-azithromycin group. This observation is not attributable to more effective malaria treatment or longer duration of prophylaxis, as treatment outcomes and incidence of malaria infections were the same in all groups. The medication was given at a high dose and has a long half-life, so recipients were potentially protected from bacterial infections for weeks at a time. The higher hemoglobin concentration may thus be due to the prevention or treatment of bacterial infection; alternatively, decreasing the bacterial burden in the gastro-intestinal tract that may lead to improved absorption of nutrients or decreased levels of chronic inflammation. Further studies will investigate the detailed etiology of this phenomenon. Azithromycin was also associated with transient neutropenia, but with no adverse clinical outcomes or long term sequelae.

There were few safety concerns. Participants in all groups experienced pruritis, as commonly occurs in dark-skinned individuals treated with chloroquine. The significantly lower rate of pruritis in the chloroquine-artesunate group was unexpected. It is possible that artesunate accelerates chloroquine metabolism, thus decreasing the adverse effects [20], [21]. Pharmacokinetic measurements are underway and may provide an explanation for this phenomenon.

### Generalizability

Several factors warrant caution before serious consideration is given to returning to chloroquine use as a wide-scale treatment policy. Although this study was large for an antimalarial drug therapeutic efficacy study, the proportion of malaria parasites under chloroquine selective pressure, relative to the entire parasite population in this community, was very small. How much drug pressure, either in total dose or duration of exposure, is required to lead to a detectable change in the distribution of drug-resistant malaria is not known. As is frequently the case in urban settings in Africa, the population was highly mobile; participants were censored from the study if they missed more than eight weeks of follow up. Losses-to-follow-up were similar across study groups, and there was no evidence to suggest that censoring was related to the outcomes of interest.

All treatments were administered under directly observed therapy, conditions that do not reflect real world use. As a result, conclusions about the efficacy of different treatment regimens and the risk of rapid selection of resistance may be different when compliance with the full treatment regimen requires the administration of two medications at home for two additional days. Moreover, while this single-site study convincingly demonstrates the absence of chloroquine-resistant falciparum malaria from this location and the immediate environs where study participants acquired malaria during the study, much larger, regional multi-site studies would be required to inform policies on using chloroquine combinations for malaria treatment or prevention.

Chloroquine-susceptible malaria has definitively returned to Malawi, and other evidence suggests the same trend is occurring throughout the region [5]. Chloroquine may have a reprised role in the treatment or prevention of malaria in the near future. With its excellent safety profile, low cost and long post-treatment prophylactic effect, it would be an attractive candidate for prevention in vulnerable groups, typically women and infants, in areas where resistance to sulfadoxine-pyrimethamine is high. It might also be considered for other groups who have not previously been targeted for malaria prevention, such as people living with HIV infection.

Any one of the combinations tested in this trial or drugs with similar pharmacodynamic characteristics might be suitable for different indications and transmission settings. The optimal selection of the partner drug(s) should be made based on knowledge of epidemiology including risk of re-infection during the post-treatment period, population pharmacokinetic characteristics, drug-resistance patterns, and other key factors related to implementation including cost, availability and ease of adherence.

## Supporting Information

Checklist S1 **CONSORT Checklist.**
(DOC)

Protocol S1 **Trial Protocol.**
(DOC)
