# Supplementary material for: A Longitudinal Trial Comparing Chloroquine as Monotherapy or in Combination with Artesunate, Azithromycin or Atovaquone-Proguanil to Treat Malaria
Source: PLoS One. 2012 Aug 17;7(8):e42284. doi: 10.1371/journal.pone.0042284 (PMC3422316; doi:10.1371/journal.pone.0042284)
Supplement: Protocol S1 — Trial Protocol. (DOC) [file pone.0042284.s002.doc]

A longitudinal study of chloroquine as monotherapy or in combination with artesunate, azithromycin or atovaquone-proguanil to treat malaria in children in Blantyre, Malawi

**Funding Mechanism:** U01-AI044824

With additional support from Pfizer

**DMID Protocol number:** 06-0022

**Principal Investigator:** Christopher Plowe, MD, MPH

Malaria Section, Center for Vaccine Development

University of Maryland School of Medicine

685 West Baltimore Street, HSF1-480

Baltimore, MD 21201 USA

Tel 410-706-2491 / 3082 Fax 410-706-1204 / 6205

Email cplowe@medicine.umaryland.edu

<http://medschool.umaryland.edu/CVD/som.html>

**IND Sponsor:** DMID

# **DMID Protocol Champion:** Philip Coyne, MD

# **DMID Medical Monitor:** Jonathon Rose

# **DMID Regulatory Affairs Specialist:** Blossom Smith

# **Draft or Version Number:** *4.0*

# **28 April 2008**

Statement of Compliance

This study will be conducted in accordance with Good Clinical Practice (GCP), including International Conference on Harmonization (ICH) Guidelines as required by U.S. Code of Federal Regulations dealing with clinical studies and investigational new drug applications (45 CFR 46 and 21 CFR including parts 50 and 56 related to informed consent and IRB regulations and 312 related to INDs). In addition, all applicable local laws and regulatory requirements relevant to the use of the study drugs will be followed

Signature Page

The signature below constitutes the approval of this protocol and the attachments, and provides the necessary assurances that this trial will be conducted according to all stipulations of the protocol, including all statements regarding confidentiality, and according to local legal and regulatory requirements and applicable US federal regulations and ICH guidelines.

| Principal Investigator: | | | |
| --- | --- | --- | --- |
| Signed: |  | Date: |  |
|  | Christopher V. Plowe, MD, MPH  Professor |  |  |

Table of Contents

Page

[Statement of Compliance ii](#__RefHeading___Toc142814923)

[Signature Page iii](#__RefHeading___Toc142814924)

[Table of Contents iv](#__RefHeading___Toc142814925)

[List of Abbreviations viii](#__RefHeading___Toc142814926)

[Protocol Summary x](#__RefHeading___Toc142814927)

[1 Key Roles 14](#__RefHeading___Toc142814928)

[2 Background Information and Scientific Rationale 18](#__RefHeading___Toc142814929)

[2.1 Background Information 18](#__RefHeading___Toc142814930)

[2.1.1 Longitudinal clinical trial of anti-malaria drug efficacy 19](#__RefHeading___Toc142814931)

[2.2 Rationale 20](#__RefHeading___Toc142814932)

[2.3 Experience with study drugs 21](#__RefHeading___Toc142814933)

[2.3.1 Mechanisms of action and resistance 21](#__RefHeading___Toc142814934)

[2.3.2 Efficacy 22](#__RefHeading___Toc142814935)

[2.3.3 Pharmacokinetics 24](#__RefHeading___Toc142814936)

[2.3.4 Safety 24](#__RefHeading___Toc142814937)

[2.4 Potential Risks and Benefits 27](#__RefHeading___Toc142814938)

[2.4.1 Known potential risks 27](#__RefHeading___Toc142814939)

[2.4.2 Known Potential Benefits 28](#__RefHeading___Toc142814940)

[3 Objectives 29](#__RefHeading___Toc142814941)

[3.1 Study Objectives 29](#__RefHeading___Toc142814942)

[3.2 Study Outcome Measures 29](#__RefHeading___Toc142814943)

[3.2.1 Primary Outcome Measures 29](#__RefHeading___Toc142814944)

[3.2.2 Secondary Outcome Measures 29](#__RefHeading___Toc142814945)

[4 Study Design 34](#__RefHeading___Toc142814946)

[4.1 Study site 34](#__RefHeading___Toc142814947)

[4.2 Description 34](#__RefHeading___Toc142814948)

[4.3 Definition of treatment outcomes 35](#__RefHeading___Toc142814949)

[4.4 Substudies 37](#__RefHeading___Toc142814950)

[4.4.1 Caretaker surveillance study. 37](#__RefHeading___Toc142814951)

[5 Study Enrollment and Withdrawal 41](#__RefHeading___Toc142814952)

[5.1 Subject Inclusion Criteria 41](#__RefHeading___Toc142814953)

[5.1.1 Rationale for Inclusion Criteria 42](#__RefHeading___Toc142814954)

[5.2 Subject Exclusion Criteria 42](#__RefHeading___Toc142814955)

[5.2.1 Rationale for Exclusion Criteria 43](#__RefHeading___Toc142814956)

[5.3 Recruitment 44](#__RefHeading___Toc142814957)

[5.4 Treatment Assignment Procedures 44](#__RefHeading___Toc142814958)

[5.4.1 Randomization Procedures 44](#__RefHeading___Toc142814959)

[5.4.2 Masking Procedures 45](#__RefHeading___Toc142814960)

[5.4.3 Reasons for Withdrawal 45](#__RefHeading___Toc142814961)

[5.4.4 Handling of Withdrawals 45](#__RefHeading___Toc142814962)

[5.4.5 Termination of Study 46](#__RefHeading___Toc142814963)

[6 Study Intervention/Investigational Product 47](#__RefHeading___Toc142814964)

[6.1 Modification of Study Intervention/Investigational Product for a Participant 48](#__RefHeading___Toc142814965)

[6.2 Study product acquisition 48](#__RefHeading___Toc142814966)

[6.3 Product Storage and Stability 49](#__RefHeading___Toc142814967)

[6.4 Accountability Procedures for the Study Intervention/Investigational Product(s) 49](#__RefHeading___Toc142814968)

[6.5 Assessment of Subject Compliance with Study Intervention/Investigational Product 50](#__RefHeading___Toc142814969)

[6.6 Concomitant Medications/Treatments 50](#__RefHeading___Toc142814970)

[6.7 Rescue therapy 50](#__RefHeading___Toc142814971)

[7 Study Schedule 51](#__RefHeading___Toc142814972)

[7.1 Screening 51](#__RefHeading___Toc142814973)

[7.2 Enrollment/Baseline 52](#__RefHeading___Toc142814974)

[7.3 Follow-up 53](#__RefHeading___Toc142814975)

[7.3.1 Cases of malaria during follow-up period 55](#__RefHeading___Toc142814976)

[7.4 Final Study Visit 56](#__RefHeading___Toc142814977)

[7.5 Early Termination Visit 56](#__RefHeading___Toc142814978)

[8 Study Procedures/Evaluations 57](#__RefHeading___Toc142814979)

[8.1 Clinical Evaluations 57](#__RefHeading___Toc142814980)

[8.2 Rescue therapy 58](#__RefHeading___Toc142814981)

[8.3 Laboratory Evaluations 58](#__RefHeading___Toc142814982)

[8.3.1 Clinical Laboratory Evaluations 58](#__RefHeading___Toc142814983)

[8.3.2 Special Assays or Procedures 59](#__RefHeading___Toc142814984)

[8.4 Molecular analysis 59](#__RefHeading___Toc142814985)

[8.5 Specimen Shipment 60](#__RefHeading___Toc142814986)

[9 Assessment of Safety 61](#__RefHeading___Toc142814987)

[9.1 Specification of Safety Parameters 61](#__RefHeading___Toc142814988)

[9.2 Methods and Timing for Assessing, Recording, and Analyzing Safety Parameters 61](#__RefHeading___Toc142814989)

[9.2.1 Adverse Events definition 61](#__RefHeading___Toc142814990)

[9.2.2 Serious Adverse Events 62](#__RefHeading___Toc142814991)

[9.2.3 Procedures to be Followed in the Event of Abnormal Laboratory Test Values or Abnormal Clinical Findings 63](#__RefHeading___Toc142814992)

[9.3 Reporting Procedures 63](#__RefHeading___Toc142814993)

[9.3.1 Reporting AEs 63](#__RefHeading___Toc142814994)

[9.3.2 Regulatory Reporting for Studies Conducted Under DMID‑Sponsored IND for SAEs 64](#__RefHeading___Toc142814995)

[9.4 Type and Duration of Follow-up of Subjects after Adverse Events 65](#__RefHeading___Toc142814996)

[9.5 Halting Rules 65](#__RefHeading___Toc142814997)

[9.6 Safety Oversight (ISM plus DSMB) 66](#__RefHeading___Toc142814998)

[10 Clinical Monitoring 67](#__RefHeading___Toc142814999)

[10.1 Site Monitoring Plan 67](#__RefHeading___Toc142815000)

[11 Statistical Considerations 68](#__RefHeading___Toc142815001)

[11.1 Study Hypotheses 68](#__RefHeading___Toc142815002)

[11.2 Sample Size Considerations 68](#__RefHeading___Toc142815003)

[11.2.1 Safety Review 69](#__RefHeading___Toc142815004)

[11.2.2 Efficacy Review 69](#__RefHeading___Toc142815005)

[11.3 Final Analysis Plan 70](#__RefHeading___Toc142815006)

[12 Source Documents and Access to Source Data/Documents 73](#__RefHeading___Toc142815007)

[13 Quality Control and Quality Assurance 74](#__RefHeading___Toc142815008)

[14 Ethics/Protection of Human Subjects 75](#__RefHeading___Toc142815009)

[14.1 Ethical Standard 75](#__RefHeading___Toc142815010)

[14.2 Institutional Review Board 75](#__RefHeading___Toc142815011)

[14.3 Informed Consent Process 75](#__RefHeading___Toc142815012)

[14.3.1 Informed Consent/Assent Process (in Case of a Minor) 76](#__RefHeading___Toc142815013)

[14.4 Subject Confidentiality 76](#__RefHeading___Toc142815014)

[14.5 Study Discontinuation 76](#__RefHeading___Toc142815015)

[14.6 Future Use of Stored Specimens 77](#__RefHeading___Toc142815016)

[15 Data Handling and Record Keeping 78](#__RefHeading___Toc142815017)

[15.1 Data Management Responsibilities 78](#__RefHeading___Toc142815018)

[15.2 Data Capture Methods 78](#__RefHeading___Toc142815019)

[15.3 Types of Data 78](#__RefHeading___Toc142815020)

[15.4 Timing/Reports 79](#__RefHeading___Toc142815021)

[15.5 Study Records Retention 79](#__RefHeading___Toc142815022)

[15.6 Protocol Deviations 79](#__RefHeading___Toc142815023)

[16 Publication Policy 81](#__RefHeading___Toc142815024)

[17 Literature References 82](#__RefHeading___Toc142815025)

[18 Appendix 89](#__RefHeading___Toc142815026)

List of Abbreviations

| AE | Adverse Event/Adverse Experience |
| --- | --- |
| ALT | Alanine aminotransaminase |
| AP | Atovaquone-proguanil |
| bp | Base pairs of DNA |
| CFR | Code of Federal Regulations |
| CIOMS | Council for International Organizations of Medical Sciences |
| CQ | Chloroquine |
| COMREC | College of Medicine Research and Ethics Committee (University of Malawi College of Medicine) |
| CRF | Case Report Form |
| DHFR | Dihydrofolate reductase |
| DMID | Division of Microbiology and Infectious Diseases, NIAID, NIH, DHHS |
| DSMB | Data and Safety Monitoring Board |
| EC | Ethics Committee |
| FDA | Food and Drug Administration |
| FWA | Federal-wide Assurance |
| GCP | Good Clinical Practice |
| HIV | Human Immunodeficiency Virus |
| IB | Investigator’s Brochure |
| ICF | Informed Consent Form |
| ICH | International Conference on Harmonisation |
| ICMJE | International Committee of Medical Journal Editors |
| IND | Investigational New Drug Application |
| IRB | Institutional Review Board |
| ISM | Independent Safety Monitor |
| JAMA | Journal of the American Medical Association |
| MR4 | Malaria Research Reference and Reagent Resource |
| MSU | Michigan State University |
| N | Number (typically refers to subjects) |
| NEJM | New England Journal of Medicine |
| NIAID | National Institute of Allergy and Infectious Diseases, NIH, DHHS |
| NIH | National Institutes of Health |
| OCRA | Office of Clinical Research Affairs, DMID, NIAID, NIH, DHHS |
| OHRP | Office for Human Research Protections |
| OHSR | Office for Human Subjects Research |
| ORA | Office of Regulatory Affairs, DMID, NIAID, NIH, DHHS |
| PCR | Polymerase chain reaction |
| PD | Pharmacodynamics |
| PI | Principal Investigator |
| PID | Participant identification number |
| PK | Pharmacokinetics |
| pfCRT | *Plasmodium falciparum* chloroquine resistance transporter |
| QA | Quality Assurance |
| QC | Quality Control |
| SAE | Serious Adverse Event/Serious Adverse Experience |
| SOP | Standard Operating Procedure |
| SP | Sulfadoxine-pyrimethamine |
| UMB | University of Maryland, Baltimore |
| US | United States |
| WHO | World Health Organization |

Protocol Summary

| **Title:** | A longitudinal study of chloroquine as monotherapy or in combination with artesunate, azithromycin or atovaquone-proguanil to treat malaria in children in Blantyre Malawi |
| --- | --- |
| **Population:** | 640 children aged 6 months to 5 years who are found to have uncomplicated symptomatic malaria in Blantyre, Malawi. |
| **Number of Sites:** | 1, Blantyre Malaria Project Research Clinic at the Ndirande Health Centre |
| **Study Duration:** | 2.5 years |
| **Subject Participation Duration:** | 1 year |
| **Description of Agent or Intervention:** | Participants will be assigned to one of four treatment arms. They will receive the same treatment at the time of enrollment and for every new episode of uncomplicated malaria for a year. All study medications will be administered orally:   - Chloroquine: 10 mg/kg on days 0 and 1, 5 mg/kg/day on day 2   Participants will be randomized to receive chloroquine alone or in combination with one of the following medications.   - Artesunate: 4 mg/kg once a day for 3 days - Atovaquone-proguanil (Malarone® or AP) once a day for 3 days as described in package insert and in section 5 - Azithromycin 30 mg/kg once a day for 3 days |
| **Objectives:** | Primary:   - Compare annual incidence of malaria clinical episodes   Secondary:   - Assess antimalarial drug efficacy at first administration, by treatment arm. - Assess antimalarial drug efficacy during subsequent episodes of malaria, by treatment arm. - Measure prevalence of chloroquine resistant parasites during the trial, by treatment arm. - Assess effect of each treatment arm on anemia at the end of study participation. - Assess safety of these drugs with repeated use - Determine the chloroquine blood levels at which chloroquine sensitive and resistant parasites are able to cause infection. - Assess the effect of population movements on the risk of malaria infection . - Assess the spatial patterns and the environmental determinants of malaria infection |
| **Description of Study Design:** | This study is a randomized, open label, longitudinal clinical trial. Participants will be identified at the time of an episode of uncomplicated malaria. After enrollment, they will be randomized to one of the four treatment arms. The treatment outcome will be assessed through a standard 28-day efficacy study. Participants will subsequently be evaluated every four weeks and encouraged to return to the study clinic any time they are ill during the course of one year. If a new episode of uncomplicated malaria is diagnosed, the participant will receive the same therapy as assigned on enrollment. PCR-corrected 28 day efficacy will be evaluated for each treatment episode. |
| **Estimated Time to Complete Enrollment:** | 1.5 years |

**Schematic of Study Design**:

640 children with uncomplicated malaria, informed consent obtained, screening completed and enrolled in the study

N=160

Treat with CQ alone

N=160

Treat with CQ plus artesunate

N=160

Treat with CQ plus azithromycin

N=160

Treat with CQ plus atovaquone-proguanil

28 days follow-up for antimalarial drug efficacy: attend clinic on days 1, 2, 3, 7, 14, 21, 28

Treat for any non-malaria illness

Completion of 12 months or decision to terminate from study?

Study termination

Randomize

Monthly examination and evaluation for any illness episode.

Does the participant have clinical malaria?

YES

NOS

Administer medication assigned at enrollment

NOS

YES

# Key Roles

| **Protocol Champion** | Phillip Coyne, MD  Division of Microbiology and Infectious Diseases  NIAID  Email: pcoyne@niaid.nih.gov |
| --- | --- |
| **Principal Investigator** | Christopher Plowe, MD, MPH  Center for Vaccine Development  University of Maryland School of Medicine  685 West Baltimore Street, HSF1-480  Baltimore, MD 21201 USA  Tel 410-706-2491 / 3082 Fax 410-706-1204 / 6205  Email cplowe@medicine.umaryland.edu |
| **Co-investigators** | Miriam Laufer, MD  Center for Vaccine Development  University of Maryland School of Medicine  685 West Baltimore Street, HSF1-480  Baltimore, MD 21201 USA  Tel 410-706-5333 / 2491 Fax 410-706-1204 / 6205  Email mlaufer@medicine.umaryland.edu  Fraction Dzinjalamala, PhD  Blantyre Malaria Project  University of Malawi College of MedicineBlantyre, Malawi  Telephone: +265-1-676-021  E-mail: [fkdz72@hotmail.com](mailto:fkdz72@hotmail.com)  Marjorie Chaponda, M.D., M.P.H.  P.O. Box 32256  Blantyre 3, Malawi, Central Africa  Tel +265-1-675-021  Mobile +265-9-920-916  Fax +265-1-670-542  E-mail: [mchaponda@bmp.medcol.mw](mailto:mchaponda@bmp.medcol.mw), [mechaponda@yahoo.com](mailto:mechaponda@yahoo.com)  Don P Mathanga, MBBS M.P.H., PhD  Malaria Alert Center,  College of Medicine,  P/Bag 360,  Chichiri,  Blantyre 3, Malawi.  Tel +265-1-870-145  Fax +265-1-676-217  Email: [dmathang@mac.medcol.mw](mailto:dmathang@mac.medcol.mw)  Matthew Laurens, MD, MPH  Center for Vaccine Development  University of Maryland School of Medicine  685 West Baltimore Street, HSF1-480  Baltimore, MD 21201 USA  Tel 410-706-5328 / 2491 Fax 410-706-1204 / 6205  Email mlaurens@medicine.umaryland.edu  Happy Phiri, Ph.D.  Malawi-Liverpool Wellcome Clinical Research Program  PO Box 30096  Blantyre 3  Blantyre, Malawi  Telephone +265-9-813915  Email [hphiri@mlw.medcol.mw](mailto:hphiri@mlw.medcol.mw)  Nicholas Connors  Blantyre Malaria Project  University of Malawi College of Medicine  Blantyre, Malawi  Telephone: +265-1-676-021  E-mail: nicholasconnors@hotmail.com |
| **Overseas Collaborator** | Terrie Taylor, DO  Blantyre Malaria Project, University of Malawi College of Medicine  Queen Elizabeth Central Hospital  Blantyre, Malawi  Telephone: +265-1-675-021  E-mail: [taylort@msu.edu](mailto:taylort@msu.edu)  -and-  Michigan State University  B315-C W. Fee Hall  Dept of Internal Medicine  College of Osteopathic Medicine  E. Lansing MI 48824 USA  Tel 517-353-8975  Fax 517-432-1062 |
| **Clinical Coordinator** | Phillip Thesing, DO  Blantyre Malaria Project, University of Malawi College of Medicine  Blantyre, Malawi  Telephone: +265-9-969-221 (mobile), +265-1-688-394 (clinic), 265-1-675-021 (project office)  E-mail: pthesing@medicine.umaryland.edu,  -and-  Center for Vaccine Development  University of Maryland School of Medicine  685 West Baltimore Street, HSF1-480  Baltimore, MD 21201 USA  Tel 410-706-2491; Fax 410-706-1204  Email: pthesing@medicine.umaryland.edu |
| **Medical Monitor** | Jonathon Rose  DMID  National Institute of Allergy and Infectious Diseases  Email rosejon@niaid.nih.gov |
| **Center for Vaccine Development Regulatory Specialist** | Karen Ball, RN, MS  Center for Vaccine Development  University of Maryland School of Medicine  685 West Baltimore Street, HSF1-480  Baltimore, MD 21201 USA  Tel 410-706-0431 Fax 410-706-6205  Email kball@medicine.umaryland.edu |
| **Independent Safety Monitor** | Dr. Peter Moons  Department of Paediatrics  Queen Elizabeth Central Hospital  Chichiri  Blantyre, Malawi  +265-9-663-007  Email pmoons@medcol.mw |

# Background Information and Scientific Rationale

## Background Information

In 1993, Malawi was the first sub-Saharan African country to discontinue the routine use of chloroquine and to elevate the antifolate combination sulfadoxine-pyrimethamine (SP) to the antimalarial of first choice nationwide. The decision was based on chloroquine's increasing failure to produce adequate clinical and hematological recovery (1). Since 1993 SP has been the only available treatment for uncomplicated malaria in all government health facilities, and is dispensed without prescription. Although chloroquine has remained available by prescription and through unauthorized private sources, a national information campaign was largely successful in convincing health practitioners and the public to accept SP as the treatment of choice for children with malaria.

It has been speculated that years of reliance on antimalarials other than chloroquine might lead to the reemergence of chloroquine-sensitive *P. falciparum* and permit the reintroduction of this safe and affordable drug. We retrospectively measured the prevalence of the *pfcrt* 76T molecular marker for chloroquine-resistant *P. falciparum* malaria in Blantyre, Malawi. From 1992 to 2000, we documented a gradual decrease in prevalence of the mutation associated with chloroquine resistance (2) and neither we nor other investigators have found any parasites with the mutation since 2001 (S. Nkhoma, personal communication and D. Bell, personal communication).

We have demonstrated the clinical efficacy of chloroquine, as well. In preparation for the present study, we conducted a trial to assess the efficacy of chloroquine in treating children with uncomplicated malaria at the Blantyre Malaria Project Research Clinic at the Ndirande Health Centre from May through December, 2005. Two hundred ten children with uncomplicated malaria were randomly assigned to receive either SP, the standard therapy in Malawi, or chloroquine. Among 105 children randomized to receive chloroquine monotherapy, only one experienced treatment failure. The results are shown in Table 1. Based on these results, we have determined that chloroquine is safe to use to treat malaria in this population.

|  | Chloroquine | SP |
| --- | --- | --- |
| Adequate clinical and parasitologic response (% per-protocol) | 79 (99) | 16 (18) |
| Early treatment failure (% per-protocol) | 1 (1) | 21 (24) |
| Late clinical or parasitologic failure (% per-protocol) | 0 (0) | 50 (57) |
| Outcomes unknown | 25 | 18 |
| Total | 105 | 105 |

Table 1: Outcomes of 28-day efficacy study of chloroquine vs. SP for the treatment of uncomplicated malaria among children in Blantyre, Malawi.

### Longitudinal clinical trial of anti-malaria drug efficacy

Current in vivo methods for measuring antimalarial drug efficacy in high-transmission areas use a 14 or 28-day follow-up period, but a single-episode study misses several critical factors in assessing the efficacy and impact of antimalarial treatment. When follow-up is extended beyond 28 days, more cases of apparent resistance or treatment failure are found (3). Single episode studies cannot assess the impact of therapy on the incidence of malaria over time.

These limitations of standard in vivo studies have led us to advocate longitudinal studies of drug efficacy (4). In addition to measuring efficacy of individual treatments, longitudinal studies measure sustained efficacy with repeated use of the same regimen over time, a scenario that more accurately reflects the real-life use of antimalarial medication. The primary outcome of interest, the incidence of malaria episodes, as well as the secondary outcomes of anemia and severe malaria, are all highly relevant to public health policy-makers, as they reflect not only the burden of disease, but also the utilization of health resources.

Longitudinal studies also permit assessment of how pharmacokinetic properties of drugs affect the incidence of treatment episodes. We collaborated on the first such longitudinal trial of antimalarial drug efficacy, a double-blinded, placebo controlled trial of SP and chlorproguanil-dapsone in Kenya and Malawi. These otherwise similar antifolate combinations are distinguished by the much shorter half-life of chlorproguanil-dapsone and its higher efficacy in Africa, where it has been shown to be highly efficacious even against infections resistant to SP (5). This study randomized children to receive one drug or the other for all uncomplicated falciparum malaria episodes over the course of a year, and compared the cumulative number of treatment episodes and rates of treatment failure and anemia (6). In Malawi, chlorproguanil-dapsone had an efficacy of 95% compared to only 80% for SP. Yet there were no differences between the treatment groups in the incidence of uncomplicated malaria episodes, anemia or severe malaria, indicating that the potential disadvantage of lacking a long prophylactic effect due to chlorproguanil-dapsone’s short half-life was offset by its better efficacy and possibly by less selection for antifolate resistant parasites. Researchers in Uganda have also used this method to demonstrate the superiority of SP plus amodiaquine over SP plus artesunate by comparing incidence density of malaria treatment episodes (7).

## Rationale

Combination therapy is becoming the mainstay of malaria treatment (8). In general, the goal of combination therapy is to treat resistant infections successfully and to prevent the emergence and spread of resistance. To date, countries have adopted combination drugs based on short term efficacy studies and availability. Optimal strategies for formulating combinations have not been established. One proposed goal of combination therapy is to combine a short-acting, highly effective medication (such as artesunate) with a longer-acting drug to offer prophylactic benefit and protect against late recrudescence. Alternatively, some have proposed matching of half-lives in the selection of drugs. The antimalarial combination therapies currently in use were not designed based on optimal pairing of drugs to deter the development and spread of parasite resistance to the individual partner drugs in settings of high malaria transmission. Careful studies are needed to identify the pharmacokinetic and pharmacodynamic properties of drug combinations that will deter resistance and prolong the useful therapeutic life of the next generation of antimalarial drug combinations. The reemergence of chloroquine-sensitive malaria in Malawi and the ability to detect chloroquine resistance at the molecular level offer a unique opportunity: we will be able to measure the emergence of chloroquine resistant parasites with repeated treatment with different combination therapies even if clinical treatment failure does not occur.

We have selected three partner drugs to use in combination with chloroquine in order to determine the pharmacokinetic and pharmacodynamic properties that most optimally protect against the emergence or re-emergence of chloroquine resistance. Artesunate is a highly effective, but very short acting, medication that will decrease parasite biomass rapidly but will no longer have any activity in the blood one day after therapy. Azithromycin is slow acting in its antimalarial activity, but remains at a high level in the blood for approximately 5-10 days after therapy, based on data from bacterial infection. It is a moderately effective antimalarial. Finally, atovaquone, in the AP coformulated Malarone, has a very prolonged terminal elimination phase and likely approximates the terminal half-life of chloroquine. Although its pharmacokinetic properties are similar to azithromycin, its pharmacodynamic properties are different, i.e., it is a more effective drug, so we expect that antimalarial activity will be maintained as the drug level gradually decreases. The specific combinations being tested may not be optimal for eventual deployment, but all combinations are likely to be safe and highly efficacious. Rather, we hope to answer the question: for how long does a partner drug need to be active to protect against resistance? The results of this study will thus inform the rational design of the next generation of combination therapies.

## Experience with study drugs

### Mechanisms of action and resistance

Chloroquine is the most widely used antimalarial drug in the world. It concentrates in the digestive vacuole of the malaria parasite where it acts by inhibiting the breakdown of heme, leading to the buildup of toxic metabolites. Chloroquine resistance occurs when a decreased level of chloroquine is able to accumulate in the parasite’s digestive vacuole. This decreased accumulation is modulated by the *Plasmodium falciparum* resistance transporter (PfCRT) , located on the membrane of the digestive vacuole (9).

Artemisinin acts through a two step process. Activation involves the iron-mediated cleavage of the endoperoxidase bridge to produce an unstable free radical, followed by alkylation, which leads to the formation of a covalent bond between the medication and the malaria protein (10). With increased oxidative stress, cytotoxicity, phagocytosis and parasite clearance are enhanced. Cytoadherence and rosetting are also inhibited, preventing the pathologic changes associated with disease (11). Clinical resistance to artemesinin derivatives has not been observed. A recent report has found an inconsistent association between PfATPase6 and an increased IC50 for artemether among field parasite isolates subjected to rapid in vitro susceptibility testing (12). The mechanism of this decrease in susceptibility is unknown.

The antimalarial activity of azithromycin has not been described fully. It is thought to target the ribosome of the malaria parasite, analogous to the established targeting of the bacterial ribosome.

Atovaquone inhibits parasite mitochondrial electron transport and proguanil inhibits parasite dihydrofolate reductase (DHFR) through its active metabolite, cycloguanil, and also through a direct mechanism outside the folate pathway that has not been well characterized (13). Both interfere with two separate pathways involved in the biosynthesis of pyrimidines which are essential for nucleic acid replication. Resistance to atovaquone can develop quickly when it is administered alone, through a point mutation in the cytochrome b gene. By changing the DHFR binding site, parasites can develop resistance to cycloguanil, but the direct anti-parasitic effect of proguanil is maintained in the presence of DHFR mutations. Proguanil was chosen to coformulate with atovaquone in the commercial product, Malarone, because proguanil has synergistic activity with atovaquone and it also decreases the parasite burden rapidly, decreasing the chance of emergence of a resistant parasite (14). Despite the ability of parasites to develop resistance to each of these drugs independently, resistance to the synergistic combination has been slow to emerge.

### Efficacy

Chloroquine was the first drug deployed in an attempt to rid the world of malaria after World War Two. It was highly effective initially, but chloroquine resistance emerged in Asia and South America and reached the African continent in the 1970s (15). As we described above, we have documented in Malawi the replacement of predominantly chloroquine resistant parasites in the 1990s by exclusively chloroquine susceptible parasites since 2000, presumed to be a result of the cessation of chloroquine use. The clinical study conducted in 2005 confirmed chloroquine efficacy in the study population.

Because chloroquine currently has a 99% cure rate in Malawi, all study treatments administered in this trial are expected to have very high initial clinical efficacy. Differences between treatment outcomes may be detectable in late recrudescence rates and/or the incidence of malaria episodes over time and other longitudinal measures of efficacy.

Artesunate is the most widely studied antimalarial compound in the literature (16). In a review of 27 randomized controlled studies of artemisinin-based combination therapy (ACT) in comparison with standard therapy, the ACT decreased treatment failure, recrudescence rate and gametocyte carriage (17). The World Health Organization recommends that all countries with endemic malaria adopt ACTs as first line antimalarial therapy (18).

Azithromycin has been used successfully as chemoprophylaxis among semi-immune adults (19;20), but has not displayed adequate efficacy when used as monotherapy. In a small, multicenter trial in India, the combination of chloroquine and azithromycin led to a 28 day parasitologic cure rate of 97%, while the rate for chloroquine alone was only 27% (21). It has also been administered successfully as a malaria prophylactic agent with efficacy of 72% in Indonesia and 83% (when administered daily) in Kenya (19;20).

The combination of atovaquone with proguanil leads to synergistic activity against even multidrug resistant parasites(22). Previous studies have administered AP, without any additional drug, for the treatment of uncomplicated malaria. The efficacy of AP among children was 100% in Thailand for children 5-12 years of age (23) and 94% in Kenya for children 3-12 years of age after 28 days of follow-up(24). In a study in Gabon, among children weighing 5-10 kg, AP had 95% efficacy at 28 days(25).

### Pharmacokinetics

|  | **Chloroquine (26)** | **Artesunate (27;28)** | **Azithromycin (29;30)** | **Atovaquone-Proguanil (31)** |
| --- | --- | --- | --- | --- |
| **Metabolite** | Desethylchloroquine (partial) | Dihydroartemesinin | None | A: None identified  P: Cycloguanil |
| **Clearance** | Hepatic and renal | Unknown | Biliary | A; Biliary  P: Renal |
| **Half-life** | 7-10 days | ≤ 1 hour | 68 hours | A: 2-3 days  P: 12-21 hours |
| **Maximum time point when drug level sufficient for antimalarial activity has been detected** | Up to 70 days | Median 8 hours but >24 has been reported | Unknown, 14 days for bacterial infection | Up to six weeks (atovaquone) |

### Safety

Chloroquine has been the most widely used antimalarial drug in the world. It is considered extremely safe. Commonly reported symptoms include headache, malaise, dizziness, blurred vision, difficulty focusing and mild gastrointestinal upset. Non-urticarial pruritis, without rash, is a problem that is more common among dark skinned patients. The symptom usually begins within the first day after the initial dose and may last up to seven days. In our study reported above, the mean duration of pruritis was1.5 days and did not prevent completion of the full course of treatment. Severe adverse reactions are extremely rare. Some are only associated with prolonged use, such as neuromyopathy with long term prophylaxis and retinopathy with high dose administration for treatment of rheumatologic diseases (total doses of 1 g/kg or prophylaxis for greater than one year). Rare cases of idiosyncratic reactions, such as erythema multiforme and bone marrow toxicity, have been reported (32).

The artemisinin derivatives, including artesunate, are the most widely studied antimalarial drugs and have been shown consistently to lead to improved efficacy when combined with a partner drug than the partner drug alone (17). A syndrome of neurotoxicity has been observed in animal models in association with intramuscular administration of artemether and arteether, both oil-soluble artemisinin derivatives. The abnormalities were found in the vestibular reflexes and auditory system at high doses with intramuscular administration of the medications only. Neuropathologic changes were found specifically in the areas of brain stem nuclei (33-42). Administration of doses of oral artesunate greater than 200 mg/kg/day have been shown to induce neurologic changes in rodents (40;41). This dose, however, is more than 50 times the usual dose administered to patients. Oral artesunate has not been linked to neuropathologic changes in experimental animals (39;41).

The extent of neurologic damage has been linked to level of dihydroartemisinin (DHA), the active metabolite of all artemisinin derivatives. Administration of artesunate, a water-soluble compound, leads to a very short-lived presence of DHA in the blood stream. In contrast, prolonged administration of the oil-based compounds, such as arteether and artemether, lead to sustained high levels of DHA that is thought to be associated with the observed neurotoxicity (32;41)*.*

Over 175 clinical trials including artemisinin based therapy have been conducted, and none has demonstrated a concerning level of toxicity (16). Most of these studies included children. In 1999, a review of over 3,000 individuals who had received therapy in northwestern Thailand revealed mild neurologic abnormalities after therapy with mefloquine or mefloquine plus artesunate, but not artesunate alone, and no subjects with any treatment regimen were found to have permanent neurological disability (43;44). The Thai study included 813 children and the mean age of the participants was under five years in 10 out of 12 African studies reviewed in the meta-analysis. Another meta-analysis of 1,869 individuals (including 424 children under five years of age) treated with the combination lumefantrine-artemether from Africa, Asia and Europe revealed no persistent neurologic abnormalities in any of the study participants (45). Two case control studies form southeast Asia, comparing brainstem evoked response, audiometry and neurologic exams of individuals who had received multiple doses of artemisinin-based therapy with controls from the same region who had never received those medications, found no detectable neurologic difference between the two groups (46;47). These studies included a total of 100 children under the age of five years and showed no evidence of neurotoxicity in the pediatric population. In addition, autopsies from humans treated with artemether who died during the course of the malaria episode did not show the characteristic changes associated with artemisinin neurotoxicity observed in animal models (48).

Artesunate has proven to be remarkably safe, with extensive use initially in Southeast Asia and now with increasing experience in Africa. In a meta-analysis of 16 randomized trials of 5,848 subjects comparing monotherapy with a commonly used agent with artesunate combination, the occurrence of serious adverse events (SAE) did not differ between artesunate combination and non-artemisinin single drug therapy (44). SAEs that have been observed during treatment with artemisinins therapy have been associated with the underlying malaria disease. Some very unusual complications have occurred with the administration of artemisinin combination therapy that were not seen in the group receiving artemisinin therapy alone: urticaria (2), seizures (5), hemoglobinuria (3) and neuropsychiatric reactions (5) (43). There have been six reported cases of allergic reaction to artemisinin therapy, with two that have required emergent therapy. The estimated risk of anaphylactic reaction is 1 in 2,833, based on the populations studied in published reports (49). Because this drug has not been approved by the U.S. Food and Drug Administration, this study will be conducted under an FDA Investigational New Drug Application (IND).

Azithromycin has been widely used in the pediatric population to treat respiratory tract infections. The suspension has been prescribed to over 80 million children. The most common side effects are: diarrhea (1.5%), abdominal pain (<5%), vomiting (2-4%), nausea (<1%) and rash (1%). The adverse event distribution associated with high dose azithromycin (20 mg/kg/day for three days) are the same as with the lower dose (10 mg/kg/day) (50). Laboratory data were similar to comparator drugs. A slight decrease in neutrophil count (500-1500/mm^3^) was noted in approximately 15% of children who received 30 mg/kg. None reached a level below 500/mm^3^. In post-marketing experience, no pediatric patients have required discontinuation of therapy due to treatment-related laboratory abnormalities (Zithromax, package insert).

The adverse effects associated with AP use were consistent with pre-treatment symptoms likely due to underlying disease, such as cough, abdominal pain and anorexia. When compared with amodiaquine, the incidence of adverse events was the same (25). The attributable adverse effects were vomiting (10%) and pruritis (6%). Among Thai adults, mild increases in transaminases occurred with initial therapy, but by day 14, the levels had equilibrated with the other arms of the study. None caused clinical illness (Malarone package insert and (51)).

### Prior experience with proposed combinations

The combination of chloroquine plus azithromycin is being studied in multiple settings under an active IND held by Pfizer Inc.  Clinical trials of this combination have been performed in India vs P.falciparum (21) and other such trials are currently in progress (Dunne, MW, personal communication).  No safety concerns have been identified to date when these drugs are used in combination, and there does not appear to be any adverse pharmacokinetic interactions (52).

The combination of chloroquine and atovaquone/proguanil has not been previously studied in vivo (Glaxo Smith Kline, personal communication).

The combination of chloroquine and artesunate was studied in a double-blind, randomized, placebo-controlled trial in Sao Tome and Principe by Gill et al in 400 children with acute uncomplicated P.falciparum (53). The combined treatment was well tolerated, no serious drug-related adverse events were observed, and the addition of AS to CQ considerably improved treatment efficacy.  Multiple clinical trials using the combination of artesunate plus a related 4-aminoquinoline, amodiaquine, have not demonstrated any adverse pharmacokinetic or pharmacodynamic interactions.  The safety profile of this combination appears to be no different than the safety of the two individual components in this regimen.

## Potential Risks and Benefits

### Known potential risks

A complete listing of all adverse drug reactions associated with the study drugs is attached in Appendix II. Finger pricks and venipuncture are associated with small risks of bleeding, hematoma and infection. To minimize this risk, the skin is cleaned with alcohol prior to puncture, sterile, unused needles and lancets will always be used and pressure will be held at the puncture site after removal of the needle or lancet. Although the quantity of blood drawn would not lead to any ill effects on the participants’ health, some adults and rarely children feel faint with phlebotomy. The risks will be minimized by having trained technicians perform the procedure. Clinicians will be available for evaluation if there is any untoward effect.

### Known Potential Benefits

The participants in this study will have access to expeditious and high quality medical care for diagnosis and treatment of malaria, as well as other illnesses. They will also be treated with an effective antimalarial therapy. The anti-malaria treatment policy in Malawi is currently undergoing revision due to the poor efficacy of SP. As of the time of the writing of this protocol, a new treatment regimen has not been announced so the earliest implementation of a new policy is unlikely to occur until late 2007. All participants in this study will receive chloroquine, which we have shown to be safe and highly efficacious in this population, either alone or in combination with another active antimalarial medication.

# Objectives

## Study Objectives

The primary objective of this study is to compare the annual incidence of malaria.

Secondary objectives are:

1. Assess the antimalarial drug efficacy at first administration, by treatment arm.
2. Assess the antimalarial drug efficacy during subsequent episodes of malaria, by treatment arm.
3. Measure the prevalence of chloroquine resistant parasites during the trial, by treatment arm.
4. Assess the effect of each treatment arm on anemia at the end of study participation.
5. Assess the safety of repeated use of the drugs in each of the study arms.
6. Determine the chloroquine blood levels at which chloroquine sensitive and resistant parasites are able to cause infection.
7. Assess the effect of population movements on the risk of malaria infection
8. Assess the spatial patterns and the environmental determinants of malaria infection

## Study Outcome Measures

### Primary Outcome Measures

- The number of clinical malaria episodes per year of follow-up.

### Secondary Outcome Measures

1. To assess the antimalarial drug efficacy at first administration, by treatment arm.

- Rate of adequate clinical and parasitologic response 28 days after initial malaria

The 28 day outcomes represent the standard assessment of antimalarial efficacy and provide important information about the short term benefits of the drug. Since we will assess the ability of combination therapy to protect against the emergence of resistance, we are most interested in clinically apparent, in vivo drug efficacy.

- The incidence of new infections and recrudescent infections after initial treatment in each treatment arm

Parasites that emerge after treatment may be classified as new infections (arising from a new exposure) or recrudescent infections (arising from the original infection). Drugs that have poor efficacy but long half-lives may protect against new infections, but fail to cure the original infection completely. In contrast, shorter acting but highly effective medications may treat the initial infection successfully, but leave the individual susceptible to new infections in areas of high transmission. Distinguishing these two mechanisms will help to elucidate the complex interaction between the drugs and the parasite population.

1. To assess the antimalarial drug efficacy during subsequent episodes of malaria, by treatment arm.

- Rate of adequate clinical and parasitologic response 28 days after subsequent new episodes of malaria
- The incidence of new infections and recrudescent infections after subsequent new episodes of malaria

The same outcomes measured after the first administration will be monitored for subsequent administrations. This ability to detect efficacy after repeated use is a strength of the longitudinal study design.

- Incidence of severe malaria

Although children who present with severe malaria will be excluded from enrollment in the study, subsequent episodes of severe disease in study participants will be assessed as study endpoints. We would expect severe malaria to be rare in this population when children are treated promptly and effectively. A significant difference in the rate of severe disease, if detected, would be an important finding.

1. To measure the prevalence of chloroquine resistant parasites during the trial, by treatment arm.

- The prevalence of parasites with the mutation *pfcrt* 76T on Day 0 of each episode of malaria
- The prevalence of parasites with the mutation *pfcrt* 76T in cases of recurrent parasitemia after therapy

Because the genetic marker for CQ resistance is well-defined, we have the unique opportunity to assess not just resistance that is manifest on the clinical level, which may be confounded by acquired immunity, but also on the molecular level.

1. To assess the effect of each treatment arm on anemia at the end of study participation.

- Hemoglobin level without parasitemia at the close of the study

Childhood anemia is multifactorial in impoverished regions in sub-Saharan Africa and leads directly and indirectly to increased morbidity and mortality. Malaria is an important contributor to this pervasive problem (54). In our effort to simulate the public health implications of various antimalarial drug policies, we will attempt to assess the effect of each treatment arm on the general well-being of the children by measuring rates of anemia at the close of the study. We will not use the hemoglobin level at the start of the study as a point of comparison because all children will be enrolled with an episode of malaria, which will often be an acute cause of anemia. We will compare the prevalence of anemia, defined as hemoglobin <8 g/dL, at the end of one year of follow-up among all four arms.

1. To assess the safety of repeated use of the drugs in each of the study arms.

- Renal and hepatic function
- Neurological examination

Some of these medications have been associated with mild, transient abnormalities in hepatic and renal laboratory tests. We will assess these side effects in the context of repeated dosing over the course of the year. In addition, we will monitor the neurologic function of the participants due to the unique concerns associated with artemisinin derivatives.

1. To determine the chloroquine blood levels at which chloroquine sensitive and resistant parasites are able to cause infection.

- Chloroquine pharmacokinetic curve for the population
- Drug concentration at the time of parasitemia

Ideally, we would like to know the drug level just prior to a detectable parasitemia in the participant since at that level, parasites were able to begin to replicate. Since it is impossible to anticipate when a new patent parasitemia will occur, we will attempt to predict the level retrospectively by obtaining the pharmacokinetic curve in the population. We will also measure the level at the time of recurrent parasitemia in case the participant was found to vary significantly from the mean.

1. Assess the effect of population movements on the risk of malaria infection.

- Risk of malaria in children who traveled outside the city compared to those who did not.

Our interest is the first incident of malaria. Malaria illness will be defined as any level of microscopy-confirmed *Plasmodium falciparum* parasitemia accompanied by signs or symptoms consistent with malaria either leading to treatment-seeking behavior or reported during follow-up visits. This definition is more inclusive since practicing physicians in a malaria endemic area would treat a person who presents complaining of illness and who had documented parasitemia. This definition will enable the longitudinal study to capture all first episodes of malaria illness that would be treated under standard medical practice. Time to malaria illness after baseline is defined as number of days without malaria illness after baseline up to the date of the first illness. Dates will be recorded for all the events occurring prior to or during follow-up.

1. Assess the spatial patterns and the environmental determinants of malaria infection

- Distribution of malaria cases in relation to environmental factors in Ndirande.

In order to evaluate whether there is clustering of cases and/or spatial correlation with environmental variables, we will carefully analyze the spatio-temporal pattern of all cases. All new malaria illnesses after the initial treatment will be escorted home and their location geo-referenced using GPS. Information will be entered into ArcGIS (*ESRI, Redland, CA*) to create a spatial point pattern. This will be linked to the database with information on health, demographic, economic, social, ecological and agricultural characteristics.

# Study Design

## Study site

Ndirande is a peri-urban hillside township adjacent to Blantyre, the largest city in Malawi. Accurate census data are not available for Ndirande, but the population is approximately 200,000. Malaria transmission is year-round, with a seasonal peak from December to March corresponding to the rainy season. The Ndirande Health Centre is the sole government health facility serving the entire township, and consists of a complex of buildings with outpatient clinics and a maternity center. Our field team of clinicians, nurses and technicians has been conducting malaria drug efficacy studies at the Health Centre continuously since 1997 and has an excellent relationship with the community. During this time more than 3,000 participants have been enrolled in five studies, including clinical trials and longitudinal studies. After operating in dedicated space within the Centre facility for several years, in 2002 we established a new research facility on the Centre grounds. The Blantyre Malaria Project Research Clinic contains rooms for clinicians and nurses, laboratory space for the microscopists, a dispensary, an administrative office, a conference room and a computer room for real time data entry. We maintain a close relationship with the Ndirande Health Centre government workers. When we provide diagnosis and treatment in the context of our clinical studies, we offer relief from overcrowded conditions in the Centre. Back-up clinical and laboratory support is available a 10 minutes drive from the Centre at the pediatric research ward in Queen Elizabeth Central Hospital.

## Description

This study is a randomized longitudinal open-label drug efficacy trial among children who present to a government health clinic with uncomplicated falciparum malaria. Otherwise healthy children who are found to have malaria and whose parents/guardians consent to participate in the study will be enrolled in this study for one year. The participants will be randomized to one of the four drug treatment arms: CQ monotherapy, CQ plus artesunate, CQ plus azithromycin, and CQ plus AP. They will continue to receive the same treatment for each new episode of malaria for the next year. If the treatment fails, participants will receive rescue therapy, but will continue in the trial. At the first diagnosis and all subsequent new episodes of malaria, a 28 day drug efficacy study will be completed. Participants will also be asked to return for routine follow-up visit every four weeks.

***Follow-up.*** With enrollment and the first dose of therapy occurring on day 0, participants will be followed actively on days 1, 2, 3, 7, 14, 21 and 28 at the study clinic, and by daily availability of a study clinician to evaluate and treat as needed even if follow-up is not scheduled. At all active and passive follow-up times, malaria smears, hemoglobin and filter paper strips will be obtained and brief history taken and physical examinations made to seek signs or symptoms of persistent or recrudescent malaria and use of any medications outside the protocol.

In addition, participants will attend four-weekly follow-up visits at the study clinic. Weight, height, hemoglobin, temperature and malaria smears and filter papers for later evaluation of asymptomatic disease will be recorded and the participant examined and questioned for signs or symptoms of malaria or other illnesses. Further evaluation, including immediate reading of the malaria smear, will take place if the parent/guardian reports symptoms, if the participant appears ill to the clinic staff or there is a documented fever. All non-malaria illnesses will be treated according to the national guidelines.

Management of early or delayed attendance of these follow up visits is detailed in the Standard Operating Procedure.

## Definition of treatment outcomes

The 2004 WHO definitions of treatment outcomes will be used as follows:

Therapeutic efficacy will be defined as:

- Early treatment failure (any one of the following)
  - danger signs or severe malaria on days 1, 2 or 3, with parasitemia. Hemoglobin less than 5 without any other signs of severe disease when a participant is receiving therapy will not be a criteria for Early Treatment Failure as hemoglobin levels frequently decrease even with effective therapy.
  - parasitemia on Day 2>Day 0 level
  - axillary temperature > 37.5^o^C on day 3 in the presence of parasitemia
  - parasitemia on day 3 > 25% of day 0 level
- Late clinical failure
  - development of danger signs, severe malaria or axillary temperature > 37.5^o^C or a history of fever within the past 24 hours in the presence of parasitemia during days 4-28
  - and no criteria for early treatment failure
- Late parasitologic failure
  - Presence of parasitemia on day 28 without fever
  - and without previously meeting any of the criteria for early treatment or late clinic failure
- Adequate clinical and parasitologic response
  - absence of parasitemia on day 28
  - and without previously meeting any of the criteria of early treatment or late clinical failure

A new episode of malaria will refer to any episode of clinical malaria (symptoms and a positive malaria smear) that occurs more than 14 days after therapy.

If a seizure meets the following criteria of a febrile seizure, the episode will not be considered a danger sign. The following criteria must be met:

1. Occurring in the presence of a reported or documented fever or with a rise in temperature.
2. Generalized seizure witnessed by reliable family member or study staff
3. Duration ≤30 minutes
4. Absence of mental status impairment two hours after the event..
5. Normal neurological examination by a study clinician or investigator within 24 hours of seizure.

## Substudies

### Caretaker surveillance study.

This has been submitted as a separate protocol to DMID, COMREC and the University of Maryland IRB.

### Pharmacokinetics substudy

Participants who develop malaria again after enrollment in the study will be eligible to participate in a pharmacokinetics substudy. In this study, we will collect two additional blood specimens to describe the pharmacokinetics profiles of the partner drugs. This understanding of the pharmacokinetic profile is an important key to understanding observed efficacy, or lack of efficacy, in each treatment arm. Participants will be randomly assigned to have blood drawn at for drug levels two time points after drug administration to capture the time to the maximum concentration (T_max_) and the maximum concentration (C_max_) and to characterize the elimination phase. In addition, we will use a portion of the serum collected during the routine venipuncture on day 0, for the collection of parasites, to measure baseline drug levels in the blood.Serum from these time points will also be used to conduct bioassays to determine the relationship between pharmacokinetics and pharmacodynamics of the drugs. At this time, we have funding to begin preliminary analysis of these specimens, through a University of Maryland Intramural New Investigator Award to Dr. Laufer. Dr. Dzinjalamala has submitted a grant to NIAID to support the full analysis of these samples. It is necessary to begin collection of these samples during the study, even if there is a delay in obtaining the funding to conduct the analysis.

Participants will be randomly assigned to two time points for additional 3 cc venous sampling based on their partner drug assignment. In the artesunate arm, levels will be measured from 30 minutes to four hours after a dose. In the azithromycin arm, levels will be measured three hours after the dose, before the second or third dose or on follow up days 3, 7, 14, 21, 28. In the atovaquone-proguanil arm, participants will be assigned to levels at one to three hours after a dose, before the second or third dose or follow up days 3, 7, 14, 21, 28. The sampling scheme for atovaquone proguanil reflects the need to capture the pharmacokinetic characteristics of both drugs.

Participation in this study will be optional for individuals who enroll in the longitudinal trial. They will be offered participation only during an episode of malaria after the first and it will be clearly explained that their decision does not affect their participation in the longitudinal trial. Although this substudy will be mentioned in the initial informed consent process, a separate informed consent process will occur when the participant is eligible for this substudy. Because the children who are enrolling in the study are acutely ill with malaria, we do not want to prolong the informed consent process to describe this portion of the study. In addition, those participants randomly assigned to chloroquine alone will not be eligible. The informed consent process will be conducted in the manner described in Section 14.

### Endothelial adhesion and malaria pathology substudy

This study is funded through a grant to Dr. Happy Phiri of the Malawi-Liverpool-Wellcome Trust Clinical Research Programme (MLW) from the Gates Malaria Partnership. This is being included as a substudy for this protocol because the specimens already being collected from this study can be used for this study and we can avoid multiple blood draws from small children. Dr. Phiri is a talented, enthusiastic post-doctoral fellow who would otherwise not have access to specimens from cases of uncomplicated malaria that he requires for his study. The University of Maryland, Blantyre Malaria Project and MLW have had a long-standing collaboration and we see our participation in this project as part of the research cooperation that we have enjoyed for many years.

The study requires parasites from well-characterized malaria episodes. The parasites collected from episodes of malaria during the longitudinal trial will serve as uncomplicated malaria parasites to compare with parasites collected from patients with severe disease. No extra specimens will be collected for this substudy and there will be no additional cost to the study. We have added a sentence to the informed consent document to reflect the use of the parasites to understand more about the pathology of malaria. This study has already been approved by the College of Medicine Research and Ethics Committee of the University of Malawi.

**Background**

Previous studies have demonstrated that nearly all patient isolates of *Plasmodium falciparum* are able to bind CD36 and that ability to bind ICAM-1 is also common. Newbold and colleagues found that approximately 80% of patient isolates were able to bind ICAM-1 (55). Other studies have shown that patient isolates may bind to multiple host receptors (56;57). Recently, we have shown that competition does occur on endothelium between different *P. falciparum* parasite lines, and that laboratory isolates could be ranked for their efficiency of binding to epithelial cells (Phiri et al., unpublished data). The key question is whether the efficiency of binding by patient isolates is related to their ability to cause disease or the efficiency at which they do so. The present study will address this key question by modelling sequestration *in vitro* using human organ-derived endothelia, and clinical *P. falciparum* isolates from children with cerebral and uncomplicated malaria, and characterised laboratory isolates. We hypothesise that *P. falciparum* isolates from patients with cerebral malaria will bind endothelia more efficiently than those from patients with uncomplicated disease.

**Methods**

**(a) Modelling sequestration using human organ-derived endothelia and clinical *P. falciparum* isolates**

A panel of 30 fresh venous blood samples (15 from cerebral and 15 from uncomplicated malaria patients), with parasitemias of 1% or more on thin blood film examination, will be collected from paediatric patients on presentation or at admission. All blood samples will be stored as multiple stabilates in liquid nitrogen and the experiments will not be performed until all the 30 samples have been collected so that the samples can be matched in our experiments for comparison. Each of the 15 patient isolates from cerebral malaria cases will be tested against all the patient isolates from uncomplicated cases in a 15 x 15 matrix in terms of their efficiency of binding to human microvascular endothelial cells (HDMEC) and human umbilical vein endothelial cells (HUVEC).

**(b) Modelling sequestration using human organ-derived endothelia and clinical and laboratory *P. falciparum* isolates**

A total of 200 *P. falciparum* patient isolates will be collected over a period of 3 years, 100 from patients with uncomplicated malaria and 100 from cerebral malaria patients. Each patient isolate will be matched with four characterised laboratory-adapted isolates. Adhesion to HUVEC and HDMEC will be investigated using *in vitro* static assays using fresh isolates only.

**Statistical analysis**

Absolute numbers of adherent parasitised red blood cells (pRBCs) from the two patient isolates being compared will be counted in 5 fields of the microslide in part (a) and in 3 fields of the coverslip in part (b). To compare the level of adhesion between paired isolates, the number of adherent pRBCs will be expressed per mm^2^. To compare the level of adhesion between experiments using different pairs of patient isolates, the number of adherent pRBCs/mm^2^ will be further expressed as the mean number of adherent pRBC/mm^2^. Statistical differences in adhesion between isolates from uncomplicated and cerebral malaria patients, in part (a), will be tested using a Student’s paired t-test. Dr. Phiri’s PhD project looked at competition between the four laboratory isolates that will be used in the current project such that their efficiency to bind endothelia was ranked. Adhesion between uncomplicated and severe patient isolates, in part (b) of this study will therefore be compared based on this information.

# Study Enrollment and Withdrawal

The study population will be drawn from children who are brought to the Ndirande Health Centre and are found to have uncomplicated malaria. Six hundred forty children will be enrolled. We expect enrollment to occur over an 18-month period so the entire study duration will be two to two and a half years, but the duration for each participant will be one year.

## Subject Inclusion Criteria

Participants must meet all of the following inclusion criteria to be eligible to participate in this study.

- Subjects aged >6 months to 5 years presenting to Ndirande Health Centre with signs or symptoms consistent with malaria including, but not limited to, one or more of the following:
  - fever at the time of evaluation (axillary temperature > 37.5^o^C by digital thermometer)
  - report of fever within the last two days
  - clinically profound anemia (conjunctival or palmar pallor)
  - headache
  - body aches
  - abdominal pain
  - decreased intake of food or fluids
  - weakness
- Weight≥ 5kg
- Positive malaria smear for *P. falciparum* mono-infection with parasite density 2,000-200,000/mm^3^.
- Planning to remain in the study area for 1 year
- Willingness to return for four-weekly routine visits, as well as unscheduled sick visits
- Parental/guardian consent for each participant

### Rationale for Inclusion Criteria

*Subjects aged >6 months to 5 years presenting to Ndirande Health Centre with signs or symptoms consistent with malaria.* This group is the target population recommended by the WHO protocol and this age group carries the highest burden of symptomatic disease. We will enroll children who exhibit any symptoms of malaria that have prompted seeking medical attention as this circumstance is the most representative of real life conditions for malaria therapy.

*Weight≥5kg.* AP is approved for children weighing greater than or equal to 5 kg.

*Positive malaria smear for P. falciparum mono-infection* with parasite density 2,000-200,000/mm^3^. Non-falciparum plasmodium species are generally susceptible to chloroquine and are not the focus of this study. Very low level parasitemia is more likely to be an asymptomatic infection with a different etiology of the presenting symptoms. It may be misleading to assess efficacy when the parasites are unlikely to be causing the illness. More than 200,000 parasites/mm^3^ may meet the criteria for severe disease and the participant should be reviewed and treated emergently and potentially referred to the hospital, rather than going through the enrollment procedures.

*Planning to remain in the study area for 1 year and willingness to return for four-weekly routine visits, as well as unscheduled sick visits.* This study requires frequent follow-up and easy access to the clinic for evaluations of every illness. Parents/guardians and children who choose to participate in the study must understand and commit to these requirements.

## Subject Exclusion Criteria

Potential participants who meet any of the following exclusion criteria at the time of evaluation for enrollment will not be eligible for enrollment in this study.

- Signs of severe malaria: One or more of the following:
  - hemoglobin ≤5 g/dL
  - prostration
  - respiratory distress
  - bleeding
  - recent seizures, coma or obtundation (Blantyre coma score < 5)
  - inability to drink
  - persistent vomiting
- Known allergy or history of adverse reaction to CQ, artesunate, azithromycin, erythromycin or AP
- Chronic medication with any antibiotic or anti malarial medication
- Previous enrollment in this study
- Alanine aminotransferase (ALT) more than 5x the upper limit of normal or creatinine greater than 3x the upper limit of normal
- Evidence of chronic disease or physical stigmata of severe malnutrition (i.e., loss of muscle mass or subcutaneous tissue, edema, or skin or hair findings consistent with severe malnutrition)

### Rationale for Exclusion Criteria

*Signs of severe malaria.* Children who are too ill to be managed at the outpatient research clinic will be referred to the hospital for management of their severe disease. Once enrolled in the study, children who develop severe malaria will be managed at the pediatric research ward at the referral hospital, but will remain in the study.

*Chronic medication with any antibiotic or antimalarial medication.* Participants who are chronically medicated with a drug that has antimalarial activity (defined as administration of a drug for more than 14 days) will have a decreased risk of acquiring malaria. This medication will interfere with our primary measure of annual incidence of malaria.

*Previous enrollment in this study.* Children who have been enrolled in the study may be infected with parasites that do not reflect the general circulating parasite genetics. Children who have completed the study or have been terminated from the study are not eligible to enroll again.

*Laboratory values more than 3-5x the upper limit of normal.* Abnormal liver and kidney function will interfere with our ability to assess the safety of the study drugs. Because the children will be acutely ill upon recruitment, moderate laboratory abnormalities may be due to acute illness, especially mildly elevated liver enzymes. Overt signs of hepatic or renal failure will be exclusion criteria (described below). Normal values for children based on studies in southern Mozambique will be used (Quinto, et al. Tropical Medicine and International Health submitted, 2006). The exact values to be used are listed in Appendix III.

*Evidence of chronic disease or severe malnutrition.* Children who have underlying illnesses may be difficult to assess for clinical cure from malaria. In addition, children with a chronic progressive disease may be mistakenly determined to have adverse effects of the drugs.

## Recruitment

Children who are brought to the Ndirande Health Centre with symptoms suggestive of malaria will be invited for evaluation at the BMP Research Clinic at the Ndirande Health Centre. There, a blood smear will be obtained and a hemoglobin level will be checked, according to standard management in Malawi. Children with uncomplicated malaria with a parasite density that may fall within the inclusion criteria and meet other eligibility criteria will be offered to participate in screening and potentially enroll in the study. Once the informed consent process has been completed and the subject’s parent/guardian has given consent, further screening and evaluation will take place, as described in the study procedures above.

## Treatment Assignment Procedures

### Randomization Procedures

The randomization scheme will be generated by the EMMES Corporation. The study statistician has no contact with participants or the study staff that cares for the participants. Treatment assignments will be generated in randomized blocks. The randomization assignment will contain a schedule for one time point to obtain chloroquine levels from day 0 through day 28. The schedule will be the same for each treatment episode. PIDs will be retired as they are assigned to subjects and not re-assigned if a participant becomes ineligible or drops out between the time of enrollment and first drug administration.

The randomization code will be kept in a secure area by the data manager in Blantyre, as well as the EMMES Corporation.

### Masking Procedures

This study will be open label.

### Reasons for Withdrawal

Participants will be removed from the study if:

- Any clinical adverse event (AE), laboratory abnormality, intercurrent illness or other medical condition occurs such that continued participation in the study would not be in the best interest of the subject
- If the participant requires chronic antimicrobial therapy. For example, children who are found to be infected with HIV will be referred for trimethoprim-sulfamethoxazole prophylaxis and anti-retroviral eligibility evaluation and removed from the study
- Participants who miss ≥ 2 consecutive routine visits will be removed from the study

Participants may withdraw voluntarily from the study at any time.

### Handling of Withdrawals

Participants who withdraw from the study with on-going AEs will be asked to return for follow-up. Specialized tracers have been designated to help to locate participants who do not return for follow-up visits. They will go to the house of the participant (directions are given at enrollment) and try to find the participant or a family member. The staff members will return to try to find any participant who missed a scheduled visit. If the participant is not available, the staff member will ask about the child’s general health, if it is known.

We anticipate some children will not complete the full year of follow-up either due to migration or voluntary withdrawal, so we have chosen to enroll an excess of participants to allow for this loss. Participants who leave the study prematurely will not be replaced.

### Termination of Study

The study may be terminated at the discretion of DMID. As described below, the DSMB may chose to recommend closure of the study after the interim analysis or at another time if new concerns arise.

# Study Intervention/Investigational Product

All medications will be shipped to Fisher BioServices and then distributed to Malawi.

| **Medication (trade name)** | **Manufacturer** | | **Source** | **Formulation** | **Dose** | **Number to receive this medication** |
| --- | --- | --- | --- | --- | --- | --- |
| Chloroquine  (Nivaquine) | Sanofi-Aventis, France | | Shipped by manufacturer | Tablet  100 mg | Days 0,1:  10 mg/kg  Day 2:  5 mg/kg  Medication will be administered to the nearest 1/4 tablet. | 640  (160 monotherapy 480 in combination) |
| Artesunate | Sanofi-Aventis  France or Guilin pharmaceuticals | | Shipped by manufacturer (Arsumax) or  Atlantic laboratories (Guilin product) | Tablet  50 mg | Days 0, 1, 2:  <10 kg ½ tablet  10-15 kg 1 tablet  16-20 kg 1-1/2 tablets  21-30 kg 2 tablets  31-40 kg 3 tablets  >40 kg 4 tablets | 160 |
| Azithromycin | Pfizer, USA or Pliva, USA | | Donated by Pfizer or purchased through Premium Health Services | Suspension  200 mg/5cc | Days 0, 1, 2:  30 mg/kg for 3 days | 160 |
| Atovaquone-proguanil  (Malarone) | GSK, USA | Purchased through Premium Health Services | | Pediatric tablet (PT): 62.5 mg/25 mg  Full strength tablet (FST):250 mg/100 mg | Days 0, 1, 2:  5-8 kg: 2 PT  9-10 kg: 3 PT  11-20 kg: 1 FST  21-30 kg: 2 FST  >30 kg: 3 FST | 160 |

## Modification of Study Intervention/Investigational Product for a Participant

The doses will not be modified.

## Study product acquisition

Atovaquone-Proquanil (Malarone) and Azithromycin (Zithromax) are both U.S. approved drugs.  The azithromycin will either be donated by Pfizer or purchased from Premium Health Services and AP will be purchased from Premium Health Services. These study drugs will be sent to the DMID Clinical Agents Repository (Fisher Bioservices, Rockville, MD) for labeling, storage, and distribution to sites.

Chloroquine sulfate (Nivaquine) and Artesunate are not U.S. approved drugs.  Nivaquine will be acquired from the manufacturer, Sanofi-Aventis France. Artesunate will be purchased from Sanofi-Aventis , or, alternatively, artesunate manufactured by Guilin Pharmaceutical Company will be purchased through Atlantic Laboratories in Thailand.  Sanofi-Aventis and Atlantic laboratories will ship the investigational drugs to the DMID Clinical Agents Repository (Fisher Bioservices, Rockville, MD) for labeling, storage, and distribution to sites.

All study drugs will be labeled for investigational use only.

## Product Storage and Stability

The chloroquine and AP tablets should be stored at 25°C, with excursions from 15-30°C permitted. The artesunate should be maintained below 25°C and should be protected from light and moisture. The azithromycin dry powder and constituted suspension should be kept at less than 30°C. The medications will be kept in an air conditioned, locked storage area that maintains a temperature at or below 25°C. A working supply of the drugs will be kept in the dispensary only during the time of the dispensing and will return to the temperature controlled storage room when dispensing is finished for the day. Reconstituted azithromycin will remain in the refrigerator in the dispensary The storage room and the refrigerator in the dispensary will have temperature monitoring with minimum/maximum thermometers read daily by a member of the staff.

All tablets will be crushed for participants who are unable to swallow pills. Tablets will be crushed according to the study standard operating procedure. We do not have specific data about the potency of crushed artesunate pills, but this method of administration is standard practice in pediatric clinical trials of malaria drug efficacy on which most published artesunate efficacy and safety data are based.

## Accountability Procedures for the Study Intervention/Investigational Product(s)

The DMID Clinical Agents Repository (Fisher Bioservices) will ship study medications to the investigational site in Blantyre, Malawi.

When it arrives at the clinic in Blantyre, the supply manager, or a trained designee, will be responsible for taking inventory of all study treatment. Each day, a nurse pharmacist will be responsible for documenting and dispensing all study medication. The study coordinator or another investigator will be responsible for reviewing inventory and dispensary medication records to be sure that all drugs are accounted for. This inventory reconciliation will take place every 4-6 weeks. Standard operating procedures will be developed to address acquisition, transportation, storage and accountability for study drugs.

## Assessment of Subject Compliance with Study Intervention/Investigational Product

All medication will be administered under direct observation on the day of enrollment and when the subject returns for follow-up on days 1 and 2. The tablets will be crushed and administered with water for participants who are unable to swallow pills.

## Concomitant Medications/Treatments

Children who are receiving chronic therapy with any antimicrobial agent will not be eligible for the study. Short term therapy with antimicrobial agents is permitted in order to treat acute infections that develop during the study period. If participants develop the need for chronic medication, they will be terminated from the study.

## Rescue therapy

Rescue therapy will be administered to children who develop clinical treatment failure within the first 14 days after a treatment episode or who are unable to complete an adequate treatment regimen due to AEs. Rescue therapy will be recorded on the CRF. Rescue therapy for uncomplicated malaria will be based on the national treatment guidelines in Malawi. For children who develop signs or symptoms of severe malaria, parenteral therapy will be administered at the Paediatric Research Ward at the hospital. Treatment that is administered by the study team or by the Paediatric Research Ward staff after complete evaluation will not lead to removal of the participant.

# Study Schedule

Refer to Appendix I for the complete study schedule.

## Screening

Prior to any study-related screening procedure, study nurses will approach potentially eligible subjects waiting to be seen by the government clinical officer at the Ndirande Health Centre. The parents/guardians of potential participants will be invited to have the child assessed by a study clinician for possible participation in the study. All of these children will undergo a finger prick for a malaria smear and hemoglobin measurement, as part of the routine care of ill children in this region and at the Ndirande Health Centre. Those who may be eligible will be offered the choice of undergoing screening to potentially enroll in the study or receiving free treatment for their current illness without participating in the study. Subjects who do not meet inclusion criteria or who are for any other reason determined to be too ill for inclusion in the study, or who decline to participate in the study, will receive standard and appropriate treatment, including transport to the Paediatric Research Ward at the Queen Elizabeth Central Hospital for parenteral therapy when indicated for severe malaria.

During the informed consent process, a study clinician will explain the study and review the informed consent form with the participant’s parent/guardian. The family will then have the opportunity to read the form and ask questions. The parent/guardian will be asked to sign the form. If the individual is unable to sign his or her name, a thumbprint will be placed on the form and another individual will be asked to sign the form as a witness. The document also allows the parents/guardians to indicate how to handle the remaining specimens after the study procedures have been completed. Any use of study samples that is outside the scope of the objectives of this protocol will be submitted for prior review and approval by the appropriate IRBs. A single informed consent document will be signed and dated for screening and enrollment. The parent/guardian will be given a copy of the consent form at the time of enrollment. The informed consent process is described in more detail in section 14.

## Enrollment/Baseline

After informed consent is obtained, a case record form including demographic information, previous medication exposure, allergy history and physical examination will be completed. The clinician will ask the parents/guardians about their plans to remain in the area and their willingness to bring the child for all scheduled and unscheduled follow-up. If the participant remains eligible, a study number will be assigned. The participant will be sent to the laboratory for venipuncture to obtain a complete blood count, ALT, creatinine, chloroquine level CBC and parasites for cryopreservation. Two attempts will be made to obtain these specimens. If the technicians are unable to obtain a sufficient specimen, the finger will be pricked to obtain 0.5 mL for a complete blood count, ALT and creatinine. All enrolled children will be escorted home where the location of the household will be recorded using GPS. At home, a questionnaire on socioeconomic status based on household assets will be administered and detailed directions of their home recorded to facilitate active follow-up.

## Drug administration

After laboratory tests have been completed and all the eligibility criteria are met, the participant can be enrolled. After enrollment and assignment of as participant ID number, the participant will be escorted to the study pharmacy. Drug assignment will be made as described in the randomization section. The drug doses are as follows:

Chloroquine 10 mg/kg on days 0 and 1; 5 mg/kg on day 2 (to the nearest ¼ tablet)

Artesunate approximately 4 mg/kg on days 0, 1 and 2

Azithromycin 30 mg/kg on days 0, 1 and 2

Atovaquone-proguanil approximately 15-20 mg/kg on days 0, 1 and 2 (as described in the study intervention section and directed in the package insert)

Participants will be observed for at least 30 minutes to monitor for adverse reactions and to make sure the medicine is not vomited. All drug administration attempts and episodes of vomiting will be recorded on the CRF. The procedure for drug administration will be as follows:

1. Administer dose of chloroquine and observe for 30 minutes.
   1. If vomiting does not occur, go to step 2
   2. If vomiting occurs after first dose, repeat dose
   3. If vomiting occurs after second dose, consult a clinician or investigator
   4. If the clinician determines that the child appears well enough to continue with oral therapy, repeat dose
   5. If third dose is vomited within 30 minutes, transport to hospital for evaluation and parenteral therapy.
      1. If this sequence of events occurs on day 0 of the enrollment illness, the participant will be removed from the study
      2. If this sequence of events occurs on day 1 or 2 of the enrollment illness or during subsequent treatment episodes, the participant will continue in the study
2. If the participant has tolerated the chloroquine and is assigned to receive combination therapy, administer the partner drug next.
   1. If vomiting does not occur, administration complete
   2. If vomiting occurs after first dose, repeat dose
   3. If vomiting occurs after second dose, consult a clinician or investigator
   4. If the clinician determines that the child appears well enough to continue with oral therapy, repeat dose
   5. If third dose is vomited within 30 minutes, do not administer drug again and note on CRF
      1. If this sequence of events occurs on day 0 of the enrollment illness, the participant will be removed from the study
      2. If this sequence of events occurs on day 1 or 2 of the enrollment illness or during subsequent treatment episodes, the participant will continue in the study. A clinician will assess if the child requires transportation to the hospital for further evaluation and therapy.

## Follow-up

Follow-up visits may be one of four types: day of new malaria episode diagnosis, drug efficacy follow-up, routine visit, unscheduled visit.

On the initial day of a new diagnosis, a venipuncture will be performed to obtain the following studies: filter paper sample for molecular analysis, CBC, ALT, creatinine, chloroquine level and specimen for cryopreservation of parasites. The total volume will be approximately 3.5-5 mL.

Drug efficacy follow-up occurs after the initial and all subsequent administrations of the study drug to treat symptomatic malaria. Visits will occur on days 1, 2, 3, 7, 14, 21, 28. Hemoglobin, malaria smears and filter papers will be obtained on each of those days and a physical examination will be completed. Specimens from a finger prick will be obtained on two occasions after day 0: on day 14 and an additional day assigned randomly at enrollment. Chloroquine level, CBC, ALT and creatinine will be measured routinely on day 14, after acute changes associated with malaria should have resolved If the participant misses the visit corresponding to the day a chloroquine level and/or CBC, ALT and creatinine arescheduled to be collected, the study team will endeavor to collect this specimen as the next visit. Drug administration will occur on days 1 (2^nd^ dose) and 2 (3^rd^ dose).If treatment failure occurs after day 3 and the parasite density is >10,000/mm^3^, blood will be collected to measure the chloroquine level and for cryopreservation of malaria parasites for further in vitro susceptibility testing. A neurologic exam will occur on day 28.

Routine visits occur every four weeks. Malaria smear and filter paper will be obtained. The malaria smear will be read at that time only if there are complaints of an illness or the clinician suspects malaria. The encounter will include an interval history, questions about off-study drug use, recent travel, bed net use and a physical examination. At routine visits 4, 8 and 13, CBC, ALT and creatinine will be obtained for safety monitoring surveillance. All blood specimens will be collected from a finger prick except for the specimens for cryopreservation which require 3-5 ml. On all other days, the amount of blood drawn from the finger prick will range from 0.05 ml (malaria smear, hemoglobin, filter paper) to approximately 0.5 ml (malaria smear, hemoglobin, filter paper, CBC, ALT, creatinine and chloroquine level).

Unscheduled visits are expected to occur numerous times during the one year of follow-up since children in this region suffer from many acute illnesses including malaria, respiratory disease and diarrhea. During unscheduled visits, the participant will be evaluated according to standardized procedures. Any signs or symptoms of malaria will prompt the collection of a malaria smear and hemoglobin measurement. If the participant is diagnosed with malaria, he or she will receive the treatment assigned on enrollment. Further evaluation and management will be undertaken by the study team according to national guidelines.

### Cases of malaria during follow-up period

If symptomatic malaria recurs within 14 days of the initiation of study treatment, rescue therapy will be given and the episode will be coded as an early treatment failure or late clinical failure. If an illness episode occurs more than 14 days after the initiation of the last treatment, it will be considered to be a new clinical episode and treated with the study drug. The case definition of uncomplicated malaria for new clinical episodes is a positive malaria smear and any of the following signs or symptoms:

- Fever measured at the clinic
- History of fever in the past 24 hours
- Vomiting
- Abdominal pain
- Poor oral intake
- Headache
- Irritability for at least 12 hours

Any participant who has danger signs or signs of severe disease (as described above) including a parasite density greater than 10% or greater than 500,000/mm^3^ will not be treated with the study drug and will be transported to Queen Elizabeth Central Hospital for further management.

### Rescue therapy

Rescue therapy refers to medication to treat malaria when it is not appropriate to give the study drug. This includes episodes of treatment failure within 14 days of the first administration of the study drug or any case of severe malaria. Rescue therapy will be administered according to the national treatment policy of Malawi for uncomplicated or severe malaria as appropriate. At this time, Malawi is in the process of selecting which artemisinin-based combination therapy will be its first line therapy. Once the decision has been made, we will follow that policy.

## Final Study Visit

The final study visit will occur on the 13^th^ routine visit unless the participant is actively enrolled in drug efficacy follow-up or an unresolved AE remains. If the participant has not yet completed the drug efficacy follow-up, the final study visit will occur on day 28 of the drug efficacy trial or when the endpoint is reached. For participants who have unresolved AEs, follow-up will continue until the AE is resolved or stabilized.

## Early Termination Visit

If early termination occurs, and the participant is willing, the procedures associated with a routine visit will be performed. In addition, CBC, ALT and creatinine will be obtained and a neurological examination will be performed if they were not done in the past four weeks. If the participant has not completed the scheduled chloroquine level ascertainment, the chloroquine level will be measured at that time, as well.

# Study Procedures/Evaluations

Refer to Appendix I for the Schedule of Events

## Clinical Evaluations

*History-comprehensive*

- The medications administered to the child in the past week
- Any underlying medical conditions
- Allergies
- Recent travel history and bed net use

*History-possible adverse drug reactions and interval*

A standardized checklist in the case report form includes symptoms of malaria, as well as most common adverse effects of the drugs being administered.

*Questioning about antimalarial use*

The parent/guardian will be asked if any drugs have been administered to the child in the interval since the last visit. The clinician will then specifically ask about the commonly administered antimalarial drugs.

*Physical examination*

The physical examination includes vital signs (temperature, heart rate, respiratory rate), weight, height and a complete physical examination that focuses on: signs of malaria, signs of non-malaria sources of fever and adverse reactions to the medications.

*Neurological examination*

A basic age-appropriate neurological examination will be conducted at the end of every episode of malaria treatment if the participant is not ill (day 28) and also at visit number 4, 8 and 13. See appendix IV.

## Rescue therapy

Rescue therapy will be administered if the participant develops early treatment failure or late clinical failure within the first 14 days after the initiation of therapy. Rescue therapy will be administered according to the national treatment guidelines in Malawi. Participants who develop signs or symptoms of severe malaria, or are deemed too ill to receive oral therapy, will be transferred to the Queen Elizabeth Central Hospital research ward for parenteral therapy.

## Laboratory Evaluations

### Clinical Laboratory Evaluations

*Malaria smears*

Both thick and thin smears will be obtained during the study. Parasite density will be quantified each time malaria smears are obtained, either at the time of the visit if the participant is ill or at a later time. Standard operating procedures are followed to assure uniform and high quality malaria smears. Thick smears are read by counting the number of parasites seen per 200 white blood cells. Parasite density for days 0 through three will be calculated based on the exact white blood cell count obtained on day 0. Otherwise, quantification will be based on an expected 8,000 white blood cells/mm^3^.Thin smears are read by counting the number of parasites seen per 500 red blood cells. Exact parasite density is calculated based on the red blood cell count. Thin smears will be used to identify the malaria species each time the diagnosis of malaria is made.

Accurate speciation and quantification will be assured based on Standard Operating Procedures. Slides will be read by at least two readers with a tie-breaker in the case of significant discrepancies. All readers have been trained in malaria diagnostic microscopy using the Malaria Research Reference and Reagent Resource (MR4) standardized slide set designed for this purpose, and, subject to continued availability of the slide set, will be evaluated annually using the slide set to assess their competency.

*Hemoglobin*

Hemoglobin measurement is obtained from the same finger stick as the malaria smear and conducted as a point-of-care test by the nurses.

*Complete blood count*

Complete blood counts will be performed in a Coulter counter. This examination required 20 microliters of blood.

*ALT and Creatinine*

These laboratory results will be obtained from a biochemistry analyzer. This examination requires 30 microliters of whole blood.

*Chloroquine level*

0.5mL specimen will be collected from a finger stick into an EDTA microtainer. The specimen will be transported to the Wellcome Trust laboratory for chloroquine level measurement by high pressure liquid chromatography. . This analysis requires 100 microliters of blood. Two hundred microliters will be taken if possible in case of mechanical malfunction or to preserve for future validation. Some specimens may also be tested in a reference laboratory at the Liverpool School of Tropical Medicine, UK*Special Assays or Procedures*

*Cryopreservation of parasites*. Parasites from the first day of a new malaria episode and any case of late parasitologic failure will be collected from participants via venipuncture. The sample will be transported to the Wellcome Trust Clinical Research Laboratory adjacent to the BMP office on the grounds of Queen Elizabeth Central Hospital in Blantyre for separation of red blood cells for cryopreservation, and storage of serum. The purpose of cryopreservation of parasites is to allow future characterization of the parasites and confirm reduced susceptibility in vitro, in the event that clinical chloroquine resistance emerges. Serum will be used to assess the parasiticidal capacity from participants who have treatment failure.

## Molecular analysis

Molecular analysis of parasite DNA will be performed in batches and results will not be available for clinical application. Wild-type chloroquine susceptible parasites are distinguished from mutant chloroquine resistant parasites by nested-PCR followed by allele specific restriction digest, following standard procedures available on our Website: <http://www.medschool.umaryland.edu/cvd/plowe.asp>.

Cases of parasitemia from day 14 through day 28 will undergo MSP-2 genotyping following the protocol published by Cattamanchi , et al (58) or an updated method if available at the time of analysis. Recrudescent infection will be defined as a PCR product from the failure day that is within 10 base pairs (bp) of a PCR product present on the initial day of infection. A new infection will be defined as a PCR product that is more than 10 bp different in size from the strain(s) present during the initial infection. Others will be labeled as indeterminate and may be investigated further.

## Specimen Shipment

Specimens will be shipped to the University of Maryland for confirmatory tests of data generated in Malawi and for other analysis requiring equipment that is not available in Malawi. Dried filter paper specimens that are not known to carry infectious agents can be transported without any special precautions. If cryopreserved parasites and sera need to be shipped to collaborating laboratories, they will be transported in liquid nitrogen dry shippers that meet all the relevant requirements and specifications.

# Assessment of Safety

## Specification of Safety Parameters

Complete blood count, ALT and creatinine will be measured every time an episode of clinical malaria occurs, at day 14 after the treatment and at approximately 3-month intervals (see study schedule). An age-appropriate neurological examination will be conducted after resolution of the initial illness, at two regular intervals, and at the last visit of the study.

## Methods and Timing for Assessing, Recording, and Analyzing Safety Parameters

### Adverse Events definition

ICH E6 Good Clinical Practice Guidelines defines an Adverse Event (AE) as any untoward medical occurrence in a patient or clinical investigation subject administered a pharmaceutical product regardless of its causal relationship to the study treatment. An AE can therefore be any unfavorable and unintended sign (including an abnormal laboratory finding), symptom, or disease temporally associated with the use of medicinal (investigational) product. The occurrence of an AE may come to the attention of study personnel during study visits and interviews or by a study recipient presenting for medical care. Any medical condition that is present at the time of the participant is enrolled will be considered a baseline condition, and not reported as an AE. However, if the severity increases, it will be recorded as an AE.

**Severity** All adverse events will be assessed by the investigator using a protocol defined grading system in Appendix III and the DMID Pediatric Toxicity Tables. For events not included in the protocol defined grading system, the following guidelines will be used to quantify severity.

- **Mild** - causes minimal discomfort and does not interfere with normal activities.
- **Moderate** - sufficiently discomforting to interfere with normal activities.
- **Severe** - incapacitating, prevents normal activities.
- **Life**-**Threatening –** immediately life threatening however death did not occur.

Changes in the severity of an AE should be documented with the maximum severity. Adverse events characterized as intermittent require documentation of onset and duration of each episode.

**Relationship to study products**: The investigator’s assessment of an AE's relationship to study drug is part of the documentation process, but it is not a factor in determining what is or is not reported in the study. If there is any doubt as to whether a clinical observation is an AE, the event should be reported. All AEs must have the relationship to study product assessed using the following terms: associated or not associated. In a clinical trial, the study product must always be suspect. To help assess relationship, the following guidelines are used.

- Associated – There is a known temporal relationship *and/or*, if re-challenge is done, the event abates with de-challenge and reappears with re-challenge *and/or* the event is known to occur in association study product or with a product in a similar class of study products
- Not Associated - The AE is completely independent of study product administration; and/or evidence exists that the event is definitely related to another etiology.

### Serious Adverse Events

**Serious Adverse Event (SAE)**: An SAE is defined as an AE meeting one of the following conditions:

- Death during the period of protocol defined surveillance
- Life Threatening Event (defined as a subject at immediate risk of death at the time of the event)
- An event requiring inpatient hospitalization or prolongation of existing hospitalization during the period of protocol defined surveillance
- Results in a persistent or significant disability/incapacity.
- Any other important medical event that may not result in death, be life threatening, or require hospitalization, may be considered a serious adverse experience when, based upon appropriate medical judgment, the event may jeopardize the subject and may require medical or surgical intervention to prevent one of the outcomes listed above. Examples of such medical events include allergic bronchospasm requiring intensive treatment in an emergency room or at home, blood dyscrasias or convulsions that do not result in inpatient hospitalization, or the development of drug dependency or drug abuse.

All SAEs will be:

- recorded on the AE/SAE CRF
- followed by a study clinician until either resolution or stabilization
- reviewed and evaluated by the study coordinator or investigator.

### Procedures to be Followed in the Event of Abnormal Laboratory Test Values or Abnormal Clinical Findings

Described above

## Reporting Procedures

The Investigator is responsible for reporting all AEs that are observed or reported during the study, regardless of their relationship to study product.

### Reporting AEs

AEs including local and systemic reactions not meeting the criteria for “serious adverse events” should be captured on the appropriate case report form. Information to be collected includes event description, time of onset, investigator assessment of severity, relationship to study product, time of resolution of the event, seriousness, and outcome. All adverse events occurring while on study must be documented appropriately regardless of relationship. All AEs will be followed to adequate resolution or stabilization.

Any medical condition that is present at the time that the subject is screened and found eligible should be recorded and be considered as baseline and not reported as an AE. The child should receive appropriate treatment and follow-up for these events. Non-eligible children will be referred for treatment at the Ndirande District Health Centre or transport to the hospital will be arranged. If a baseline condition deteriorates at any time during the study, it should be recorded as an AE.

### Regulatory Reporting for Studies Conducted Under DMID‑Sponsored IND for SAEs

SAEs will be reported as described in the table below

|  | **DMID via PPD**  **(sponsor)** | **FDA** | **COMREC** | **University of Maryland** | **Pfizer**  For participants who received azithromycin in the past 28 days |
| --- | --- | --- | --- | --- | --- |
| **Contact information** | Medical Affairs/Pharmacovigilance  PPD Inc.  SAE Fax line 001 910 772 7069 or via the internet  dmidpvg@wilm.ppdi.com | DMID to contact | Hand delivery or email (comrec@medcol.mw) | Via BRAAN system | Kodjo Soroh, PCO Safety Head, Africa  [kodjo.soroh@pfizer.com](mailto:kodjo.soroh@pfizer.com)  or  Fax 234 1 270 3924-5 |
| **Death or a life threatening event** | 24 hours | 7 days | Within 24 hours | Within 48 hours | Immediately |
| **SAE (not included above) that is related and unexpected** | Within 72 hours | Within 15 days | Within 24 hours | Within 48 hours | Within 24 hours |
| **Study drug (not included above) related SAEs** | Within 72hours | Reported annually | Within 72 hours | Annual summary | Within 24 hours |
| **Other SAEs** | Within 72hours | Annual summary | Within 72 hours | Annual summary | Within 24 hours |
| **AEs (not SAEs)** | Annual summary* | Annual summary* | Annual summary* | Annual summary* | End of study report |

.* Unless otherwise requested by the ISM, the sponsor, the FDA or the investigators.

Other supporting documentation of the event may be requested by the pharmacovigilance contractor and should be provided as soon as possible.

All SAEs will be followed until satisfactory resolution or until the Principal Investigator or co-investigator deems the event to be chronic or the subject to be stable.

## Type and Duration of Follow-up of Subjects after Adverse Events

Participants will be followed for a total of one year, so many AEs will resolve during active enrollment in the study. AEs that remain unresolved at the end of the participants enrollment in the study will be followed until their resolution or until they have stabilized.

## Halting Rules

The data safety monitoring board (DSMB, described below) will meet after the initial follow up period for the first 150 participants and then at least every year thereafter. The data to be reviewed at the interim analysis meeting will include safety and efficacy results. Safety data will be presented as line lists of SAEs with descriptions and relatedness to study drug, as well as tallies of grade 3 and 4 AEs by treatment arm. An excess of SAEs in one arm may lead the DSMB to recommend suspending one or more arms of the study, to request further data or to require another safety analysis at a specified interval. Efficacy data will be presented including results through day 28 for all subjects in the review group. Less than or equal to 75% adequate clinical response at day 28 will lead to a discussion in the DSMB about suspension of that arm of the trial. This number alone, however, will not require stopping the study. The efficacy of all study arms is likely to be very high. This plan is subject to review by the malaria DSMB and the schedule may be changed according to their recommendation.

All SAEs will be reviewed by the independent safety monitor (ISM). The ISM may request an *ad hoc* meeting of the DSMB after the occurrence of any SAE that is associated with the study drug. Reviews by the IRB, the sponsor or the FDA may also result in suspension of further enrollment or participation.

## Safety Oversight (ISM plus DSMB)

Safety oversight will be under the direction of the Malaria DSMB established by DMID. The ISM, a local pediatrician who is not associated with the study, will review all SAEs and make periodic checks of grade 3 and 4 AEs. The ISM may request an ad hoc DSMB meeting at any time, and is encouraged to do so if an SAE occurs that the ISM deems associated with the study drug. The DSMB will operate under the rules of a DMID-approved charter that will be written and approved at the organizational meeting of the DSMB. The DSMB will meet after the first 150 participants reach the 28^th^ day after the first episode of malaria. They will then meet at least annually. At this time, each element that the DSBM needs to assess will be clearly defined. The DSMB will advise DMID of its findings.

# Clinical Monitoring

## Site Monitoring Plan

Site Monitoring will be conducted to ensure that human subject protection, study procedures, laboratory procedures, study intervention administration, and data collection processes are of high quality and meet sponsor, GCP/ICH, and regulatory guidelines, and that the study is conducted in accordance with the protocol and sponsor SOPs. DMID, the sponsoring agency, or its designee will conduct site monitoring visits as detailed in the monitoring plan or in the Manual of Procedures.

Site visits will be made at standard intervals as defined by DMID and may be made more frequently, as directed by DMID. Monitoring visits will include, but are not limited to, review of regulatory files, accountability records, case report forms, informed consent forms, medical and laboratory reports, and protocol compliance. Study monitors will meet with the study coordinator and/or investigators to discuss any problems and actions to be taken and document visit findings and discussions.

# Statistical Considerations

## Study Hypotheses

The study is powered to compare each combination individually with chloroquine monotherapy. The power is restricted to pairwise comparisons. The goal is to identify superiority of the combination arm in comparison with the monotherapy arm.

Primary objective:

Null hypothesis: Chloroquine monotherapy for treatment of malaria leads to the same annual incidence of malaria episodes as treatment with chloroquine combined with one of the partner study drugs.

Alternative hypothesis: The annual incidence rates of malaria episodes will differ between the group treated with chloroquine monotherapy and the group treated with chloroquine combined with a partner study drug.

## Sample Size Considerations

In a previous longitudinal study of malaria in this population, we found that the mean annual incidence of malaria was approximately 2.5 episodes per year (59) with a standard deviation of approximately 2. Chloroquine is presently highly effective in treating malaria and has a prolonged prophylactic effect, so we have estimated a mean rate of 1.5 malaria episodes per year with chloroquine alone. We derived the sample size required to detect a decrease in the mean to 1.0 episodes per year assuming a Poisson distribution and using a 2 sided test with alpha= .05/3=.0166. The test size was adjusted to accommodate the 3 treatment versus control comparisons. With the above requirements and for a 2 treatment contrast, 120 subjects with complete observation are required per treatment arm to have 90% power (PASS 2005). To account for migration and other causes of attrition, we will enroll an additional 33% (40 subjects) in each regimen. We will accrue 160 per arm for a total of 640 subjects.

Because of the currently estimated efficacy of chloroquine alone in Malawi (in excess of 95%), we are unlikely to determine differences in initial administration (day 28) efficacy between the treatment arms. Power is limited as all arms should have very few early failures. Rates of anemia (<8 mg/dl) at the end of one year should be available in ~120 subjects per arm. The 95% CI half-width for the difference in anemia rates, calculating the variance with a rate of .2, is approximately .10. Thus, the selected sample size should be informative about larger treatment arm differences in anemia rates.

To determine the population pharmacokinetic curve for chloroquine, we have designed a randomized unbalanced study. All participants will have chloroquine levels determined prior to drug administration, at day 14 and at one additional randomly assigned time point. The assignment will be weighted to increase measurement on day two. Planned Interim Analyses

The DSMB will decide when it would like to conduct the interim analysis. The data to be reviewed at this meeting will include safety and efficacy data.

### Safety Review

Safety data will be presented as line lists of SAEs with descriptions and relatedness to study drug as well as tallies of grade 3 and 4 AEs by treatment arm. An excess of SAEs in one arm may lead the DSMB to recommend suspending the study, to request additional data or to request another safety analysis at a specified interval.

### Efficacy Review

The study examines treatment efficacy over the course of a year. As longer term data will not be available at the interim review, the interim review will focus on initial treatment administration and 28-day clinical treatment outcomes. Less than or equal to 75% adequate clinical response at day 28 will lead to a discussion in the DSMB about suspension of that arm of the trial, although this circumstance will not constitute a formal halting rule. The efficacy of all study arms is likely to be very high as all treatment arms contain full dose chloroquine whose short-term efficacy alone is expected to exceed 95%.

## Final Analysis Plan

The final analysis of the primary objective will be a comparison of incidence rates of malaria treatment episodes per year of follow-up (not including the first infection occurring at enrollment) in each arm of the study. The rates and 95% confidence intervals will be calculated using Poisson distribution. Reported antimalarial drug use without documented positive malaria smear will not count as a malaria treatment episode. Malaria episodes treated with non-study medicines at Queen Elizabeth Central Hospital with verified positive malaria smears will be counted as malaria treatment episodes, and any such treatments will be compared among groups to rule out bias due to different rates of non-study treatment. Withdrawal of participation is described above and includes participants who miss ≥ 2 consecutive routine follow-up visits. For the primary study endpoint, follow-up time for participants while they are enrolled in the study will be included in the analysis. For participants who were withdrawn after missing > 2 consecutive follow-up visits, time after the last visit will be censored from incidence analysis.

Drug efficacy for the first and subsequent episodes of malaria will be compared using Kaplan-Meier 28-day survival estimated and compared using Cox proportional hazards regression. Per-protocol analysis will be used to compare treatment efficacy, with data excluded from treatment episodes for which treatment outcome cannot be determined. Survival analysis will also be used to assess time to first resistant infection in each arm. Categorical data including episodes of liver, kidney or neurological toxicity and presence of anemia will be compared using χ^2^ analysis. Logistic regression will be used to adjust for covariates.

Survival analysis will be used to assess the role of travel outside of Blantyre and bed net use on the risk for malarial and time to first malaria episode. This analysis will be conducted both with and without the inclusion of treatment arm as a covariate in the analysis.

Data will be entered in to the database on the computers at the field site. On a daily basis, the data will be transferred via the internet to the EMMES corporation web-based system.

### Spatial analysis

Digital base maps (topographic and features) of Ndirande will be purchased from the National spatial data center of the Malawi Geographical Information Council (MAGIC). The base maps will provide main features of the study area such as road networks (type and pattern of roads) and waterways (community water sources, rivers and drainages) as well as administrative boundaries. They will also be linked to the database containing field data. We will also use the base maps to order the satellite imagery.

Highest resolution satellite imagery will be required to map surface features of the study area. This will allow capturing of all potential breeding spaces as well as land use and land cover within the study. This will not only complement the information provided on field surveys, but also facilitate the understanding of spatial distribution of malaria risk in Ndirande. It will also provide information on environmental disturbance in the area not captured by the field surveys and base maps.

At the moment, the highest resolution available satellite imagery is the 1-metre panchromatic provided by IKONOS (*Spacing Inc*) satellite. This should provide means of extracting and distinguishing relevant geographic information covering the study areas such as road networks, houses and open grounds, waterways (rivers and streams), ponds and swamps. Ground truthing will be done to validate the satellite images. In addition, data on elevation, levels of drainage around Ndirande will be mapped and geo-referenced using GPS.

The satellite images will be processed in ERDAS (*ERDAS Imagine, Atlanta, USA*). The information extracted will then be linked to the base maps and databases in ArcGIS. Proximity of the location of houses to a river or a stream and other putative sources of malaria transmission will be identified using buffer layers which will be created along, pools of water, rivers and streams. The percentage of buffer area to the total area in a village will be used as a covariate representing proximity to rivers/streams.

Spatial Cluster analysis will be used to identify an unusually high occurrence of morbidity that is clustered in space and time. In order to identify disease clusters in smaller areas located near a potential high risk factor (putative source: ponds, rivers, water pools, streams, swamps), focused spatial cluster analysis will be carried out to assess the presence of a statistically significant grouping of cases. A number of methods exist to reveal clusters of high and low malaria rates observed during the study period. Here we propose use of Kulldorff’s SaTScan statistic**.** SaTScan uses a replication method to create a spatial scan statistic by imposing a circular window, which is then centered around each of several possible centroids positioned throughout the study region. For each centroid, the radius of the window varies continuously in size from zero to an upper limit, thus creating an infinite number of distinct geographical circles, each being a possible candidate for a cluster. In addition, a recent local indicator of spatial autocorrelation (LISA), Getis G^*^_i_, statistic, will be used to detect hotspots of malaria illnesses and relate them to putative sources of malaria transmission *.*G_i_* measures spatial association by using distance under the assumption that the variable has a natural origin, and would supplement the Kulldorff’s SaTScan.

# Source Documents and Access to Source Data/Documents

Appropriate medical and research records will be maintained for this trial, in compliance with ICH E6 GCP, Section 4.9 and regulatory and CVD requirements for the protection of confidentiality of subjects. Study investigators and the clinical coordinator will have access to all study records. Study nurses and clinicians will have access to laboratory source documents and CRFs of participants who are being actively enrolled and followed. Laboratory staff will have access to laboratory source documents. Data entry clerks and the Data Manager will have access to anonymized CRFs. Authorized representatives of DMID will be permitted to examine (and when required by applicable law, to copy) clinical records for the purposes of quality assurance reviews, audits and evaluation of the study safety and progress.

Standard case record forms (CRF) will be provided for each participant. Participants will be identified by the Participant Identification Number (PID). All data on the CRF will be recorded in black ink legibly. A correction will be made by striking through the incorrect entry with a single line and entering the correct information adjacent to it. The correction will be initialed and dated by the individual who makes the change. Any requested information that is not obtained as specified in the protocol should have an explanation noted on the CRF as to why the required information was not obtained.

The central source document will be the CRF, which includes history, physical examination, laboratory data, and diagnosis as determined by a study physician. This document will be completed for all participants. Termination forms will be recorded for all participants who leave the study, indicating the reason for exit. Laboratory tests will be labeled with PIDs. Results from the laboratory at the Health Centre and at Queen Elizabeth Hospital will be recorded in laboratory source books. The results will also be given to the clinicians for review and to record in the CRF. The original documentation of laboratory results will be kept in the participant’s file.

# Quality Control and Quality Assurance

Standard operating procedures (SOPs) will be in place for quality management including how the data will be evaluated for compliance with protocol, which documents will be reviewed and methods of training staff.

The study will be conducted at a single center, the Blantyre Malaria Project Research Clinic at Ndirande Health Centre in Blantyre, Malawi.

Site monitoring will be conducted by a DMID contractor to assure protocol compliance, ethical standards, regulatory compliance and data quality.

Following written SOPs, the monitors will verify that the clinical trial is conducted and data are generated, documented (recorded), and reported in compliance with the protocol, GCP, and the applicable regulatory requirements. Reports will be submitted to DMID on monitoring activities.

We will provide direct access to source data/documents, and reports for the purpose of monitoring and auditing by the sponsor, and inspection by local and regulatory authorities.

The EMMES Corporation will implement quality control procedures beginning with the data entry system and generate data quality control checks that will be run on the database. Any missing data or data anomalies will be communicated to the site for clarification/resolution.

# Ethics/Protection of Human Subjects

## Ethical Standard

The investigator will ensure that this study is conducted in full conformity with the principles set forth in The Declaration of Helsinki and according to the US national requirements codified in 45 CFR 46, 21 CFR 50&56 and consistent with ICHE6(R1).

## Institutional Review Board

This protocol, the informed consent document and any subsequent modifications will be reviewed and approved by the Institutional Review Board or Ethics Committee responsible for oversight of the study including the University of Malawi College of Medicine Research and Ethics Committee (COMREC), University of Maryland and Michigan State University. All of these IRBs hold valid US Federalwide Assurances from OHRP. COMREC will be the IRB of record for this study. Any amendments to the protocol or consent materials must be approved by all three IRBs before they are implemented.

## Informed Consent Process

Informed consent is a process that is initiated prior to the parents/guardians agreeing to their children’s participation in the study and continuing throughout the individual’s study participation. Discussion of risks and possible benefits will be provided to the family of the participants. Consent forms describing in detail the study interventions, procedures and risks are given to the parents/guardians. Written informed consent will be obtained from the parent/guardian prior to screening and enrollment. A copy of the consent forms will be given to the parent/guardian. Consent forms will be available in both English and Chichewa, the local language. Consent forms will be IRB-approved and the parent/guardian will be asked to read and review the document. For participants who cannot read, the complete document will be read aloud to them. Upon reviewing the document, the investigator will explain the research study to the parent/guardian and answer any questions that may arise. The parent/guardian will be asked to sign and date the informed consent document after he/she has had sufficient time to make a decision. Those who cannot write their names will be asked to indicate their consent with a thumbprint. An impartial witness should observe this process, in the case of the use of a thumbprint in place of a signature, and sign and date the informed consent document. If no impartial witness is available, a member of the study staff other than the person administering consent may serve as a witness and sign the document. The parent/guardian may withdraw consent at any time throughout the course of the trial. Any use of the study samples that is outside the scope of the objectives of this protocol will be submitted as a new protocol to all participating IRBs.

### Informed Consent/Assent Process (in Case of a Minor)

Assent will not be obtained as the participants will all be under the age of 6 years.

## Subject Confidentiality

Subject confidentiality is held in strict trust by the investigators, the staff and the study sponsor. This confidentiality is extended to cover testing of biological samples in addition to the clinical information relating to participants. The study protocol, documentation, data and all other information generated will be held in strict confidence. No information concerning the study or the data will be released to any unauthorized third party without prior approval of the sponsor.

The study monitor, regulatory authorities, IRBs or other authorized representatives of the sponsor may inspect all documents and records required to be maintained by the investigator, including but not limited to, medical records and pharmacy records. The clinical study staff will permit access to such records.

## Study Discontinuation

If the study is discontinued, participants who have not completed the three days of therapy for an episode of malaria will be given the first line malaria treatment that complies with national guidelines in Malawi. Participants will be referred to the Ndirande Health Centre or Queen Elizabeth Central Hospital for any further medical concerns.

## Future Use of Stored Specimens

Filter paper specimens, cryopreserved parasites and serum will be maintained after the study is complete if the parent/guardian has agreed to this storage during the informed consent process and indicated permission on the written document. Any analysis of these specimens that is outside the scope of the objectives of this protocol, including human genetic analysis, will be submitted for prior review and approval by the appropriate IRBs. Parasites and their genetic material may be delinked for further analysis, without any identifying clinical information.

# Data Handling and Record Keeping

The principal investigator is responsible to ensure the accuracy, completeness, legibility and timeliness of the data reported. All source documents will be completed in a neat, legible manner to ensure accurate interpretation of data. Black ink is required to ensure clarity of reproduced copies. When making changes or correction, the original entry will be crossed out with a single line and the change will be dated and initialed. Records will be kept in locked files.

## Data Management Responsibilities

All source documents and laboratory reports will be reviewed by the study team and data entry staff, to ensure that they are accurate and complete. Adverse events must be graded, assessed for severity and causality, and reviewed by the PI, an investigator, or the clinical coordinator. Data collection is the responsibility of the clinical trial staff at the BMP-Ndirande Research Clinic under the supervision of the clinical coordinator and the investigators. During the study, the investigator must maintain complete and accurate documentation for the study. The EMMES Corporation will serve as the Statistical and Data Coordinating Center for this study and will be responsible for the data management, quality review, analysis, and reporting of the study.

## Data Capture Methods

Clinical and laboratory data will be collected on case report forms and entered into a 21 CFR Part 11-compliant web-based database system designed by EMMES. The data system includes password protection and internal quality checks to identify data that appear inconsistent, incomplete or inaccurate.

## Types of Data

The types of data for this study include clinical outcomes, safety parameters and molecular analyses.

## Timing/Reports

The DSMB will decide the appropriate time for the interim analysis. The report will be produced that will include safety and efficacy. Safety data will be presented as line lists of SAEs with descriptions and relatedness to study drug as well as tallies of grade 3 and 4 AEs by treatment arm. Efficacy data will be presented as 28-day clinical treatment outcomes.

## Study Records Retention

Study documents will be retained for a minimum of 5 years after completion of the study. No formal marketing applications are expected to result from this study. No records will be destroyed without the written consent of the sponsor.

## Protocol Deviations

A protocol deviation (PD) is any noncompliance with the clinical trial protocol, Good Clinical Practice (GCP), or protocol-specific Manual of Procedures requirements. The noncompliance may be either on the part of the subject, the investigator, or the study site staff. As a result of deviations, corrective actions are to be developed by the site and implemented promptly.

These practices are consistent with Good Clinical Practice:

4.5 Compliance with Protocol, sections 4.5.1, 4.5.2, and 4.5.3

5.1 Quality Assurance and Quality Control, section 5.1.1

5.20 Noncompliance, sections 5.20.1, and 5.20.2.

It is the responsibility of the site PI/study staff to use continuous vigilance to identify and report deviations within 5 working days of identification of the PD. All protocol deviations will be addressed in study subject source documents. Protocol deviations forms will be completed by the clinical coordinator or another investigator and inputted into the data entry system and reported on a weekly basis by EMMES. A completed copy of the Protocol Deviation Form must be maintained in the Regulatory File, as well as in the subject’s source document. Protocol deviations must be sent to the local IRB/IEC per their guidelines. The site PI/study staff is responsible for knowing and adhering to their IRB requirements.

# Publication Policy

Following completion of the study, the investigator may publish the results of this research in a scientific journal. The International Committee of Medical Journal Editors (ICMJE) member journals have adopted a trials-registration policy as a condition for publication. This policy requires that all clinical trials be registered in a public trials registry such as [ClinicalTrials.gov](http://www.clinicaltrials.gov/), which is sponsored by the National Library of Medicine. Other biomedical journals are considering adopting similar policies. It is the responsibility of DMID to register this trial in an acceptable registry. Any clinical trial starting enrollment after 01 July 2005 must be registered either on or before the onset of subject enrollment. For trials that began enrollment prior to this date, the ICMJE member journals will require registration by 13 September 2005 before considering the results of the trial for publication.

The ICMJE defines a clinical trial as any research project that prospectively assigns human subjects to intervention or comparison groups to study the cause-and-effect relationship between a medical intervention and a health outcome.

# Literature References

Reference List

1. Bloland PB, Lackritz EM, Kazembe PN, Were JB, Steketee R, Campbell CC. Beyond chloroquine: implications of drug resistance for evaluating malaria therapy efficacy and treatment policy in Africa. J.Infect.Dis. 1993;167:932-7.

2. Kublin JG, Cortese JF, Njunju EM, Mukadam RA, Wirima JJ, Kazembe PN, Djimde AA, Kouriba B, Taylor TE, Plowe CV. Reemergence of chloroquine-sensitive Plasmodium falciparum malaria after cessation of chloroquine use in Malawi. J.Infect.Dis. 2003 Jun 15;187(12):1870-5.

3. Dorsey G, Njama D, Kamya MR, Cattamanchi A, Kyabayinze D, Staedke SG, Gasasira A, Rosenthal PJ. Sulfadoxine/pyrimethamine alone or with amodiaquine or artesunate for treatment of uncomplicated malaria: a longitudinal randomised trial. Lancet 2002 Dec 21;360(9350):2031-8.

4. Plowe CV. Antimalarial drug resistance in Africa: strategies for monitoring and deterrence. Curr.Top.Microbiol.Immunol. 2005;295:55-79.

5. Mutabingwa T, Nzila A, Mberu E, Nduati E, Winstanley P, Hills E, Watkins W. Chlorproguanil-dapsone for treatment of drug-resistant falciparum malaria in Tanzania. Lancet 2001 Oct 13;358(9289):1218-23.

6. Sulo J, Chimpeni P, Hatcher J, Kublin JG, Plowe CV, Molyneux ME, Marsh K, Taylor TE, Watkins WM, Winstanley PA. Chlorproguanil-dapsone versus sulfadoxine-pyrimethamine for sequential episodes of uncomplicated falciparum malaria in Kenya and Malawi: a randomised clinical trial. Lancet 2002 Oct 12;360(9340):1136-43.

7. Dorsey G, Njama D, Kamya MR, Cattamanchi A, Kyabayinze D, Staedke SG, Gasasira A, Rosenthal PJ. Sulfadoxine/pyrimethamine alone or with amodiaquine or artesunate for treatment of uncomplicated malaria: a longitudinal randomised trial. Lancet 2002 Dec 21;360(9350):2031-8.

8. World Health Organization. Antimalarial drug combination therapy: Report of a WHO techinical consultation. Geneva: World Health Organization; 2001. Report No.: WHO/CDS/RBM/2001.35.

9. Bray PG, Martin RE, Tilley L, Ward SA, Kirk K, Fidock DA. Defining the role of PfCRT in Plasmodium falciparum chloroquine resistance. Molecular Microbiology 2005;56(2):323-33.

10. Meshnick SR. The mode of action of antimalarial endoperoxides. Trans.R.Soc.Trop.Med.Hyg. 1994 Jun;88 Suppl 1:S31-S32.

11. Hien TT, White NJ. Qinghaosu. Lancet 1993 Mar 6;341(8845):603-8.

12. Jambou R, Legrand E, Niang M, Khim N, Lim P, Volney B, Ekala MT, Bouchier C, Esterre P, Fandeur T, et al. Resistance of Plasmodium falciparum field isolates to in-vitro artemether and point mutations of the SERCA-type PfATPase6. Lancet 2005 Dec 3;366(9501):1960-3.

13. Fidock DA, Nomura T, Wellems TE. Cycloguanil and Its Parent Compound Proguanil Demonstrate Distinct Activities against Plasmodium falciparum Malaria Parasites Transformed with Human Dihydrofolate Reductase. Mol Pharmacol 1998 Dec 1;54(6):1140-7.

14. Olliaro P. Mode of action and mechanisms of resistance for antimalarial drugs. Pharmacol.Ther. 2001 Feb;89(2):207-19.

15. Wellems TE, Plowe CV. Chloroquine-resistant malaria. J.Infect.Dis. 2001 Sep 15;184(6):770-6.

16. Myint HY, Tipmanee P, Nosten F, Day NP, Pukrittayakamee S, Looareesuwan S, White NJ. A systematic overview of published antimalarial drug trials. Trans.R.Soc.Trop.Med.Hyg. 2004 Feb;98(2):73-81.

17. International Artemisinin Study G. Artesunate combinations for treatment of malaria: meta-analysis. The Lancet 2004 Jan 3;363(9402):9-17.

18. World Health Organization, UNICEF. World Malaria Report 2005. 2005. Geneva, World Health Organization.
Ref Type: Generic

19. Andersen SL, Oloo AJ, Gordon DM, Ragama OB, Aleman GM, Berman JD, Tang DB, Dunne MW, Shanks GD. Successful double-blinded, randomized, placebo-controlled field trial of azithromycin and doxycycline as prophylaxis for malaria in western Kenya. Clin.Infect.Dis. 1998 Jan;26(1):146-50.

20. Taylor WR, Richie TL, Fryauff DJ, Picarima H, Ohrt C, Tang D, Braitman D, Murphy GS, Widjaja H, Tjitra E, et al. Malaria prophylaxis using azithromycin: a double-blind, placebo-controlled trial in Irian Jaya, Indonesia. Clin.Infect.Dis. 1999 Jan;28(1):74-81.

21. Dunne MW, Singh N, Shukla M, Valecha N, Bhattacharyya PC, Dev V, Patel K, Mohapatra MK, Lakhani J, Benner R, et al. A multicenter study of azithromycin, alone and in combination with chloroquine, for the treatment of acute uncomplicated Plasmodium falciparum malaria in India. J.Infect.Dis. 2005 May 15;191(10):1582-8.

22. Canfield CJ, Pudney M, Gutteridge WE. Interactions of atovaquone with other antimalarial drugs against Plasmodium falciparum in vitro. Exp.Parasitol. 1995 May;80(3):373-81.

23. Sabchareon A, Attanath P, Phanuaksook P, Chanthavanich P, Poonpanich Y, Mookmanee D, Chongsuphajaisiddhi T, Sadler BM, Hussein Z, Canfield CJ, et al. Efficacy and pharmacokinetics of atovaquone and proguanil in children with multidrug-resistant Plasmodium falciparum malaria. Trans.R.Soc.Trop.Med.Hyg. 1998 Mar;92(2):201-6.

24. Anabwani G, Canfield CJ, Hutchinson DB. Combination atovaquone and proguanil hydrochloride vs. halofantrine for treatment of acute Plasmodium falciparum malaria in children. Pediatr.Infect.Dis.J. 1999 May;18(5):456-61.

25. Borrmann S, Faucher JF, Bagaphou T, Missinou MA, Binder RK, Pabisch S, Rezbach P, Matsiegui PB, Lell B, Miller G, et al. Atovaquone and proguanil versus amodiaquine for the treatment of plasmodium falciparum malaria in African infants and young children. Clin Infect Dis 2003 Dec 1;37(11):1441-7.

26. Ducharme J, Farinotti R. Clinical pharmacokinetics and metabolism of chloroquine. Focus on recent advancements. Clin.Pharmacokinet. 1996 Oct;31(4):257-74.

27. Bethell DB, Teja-Isavadharm P, Cao XT, Pham TT, Ta TT, Tran TN, Nguyen TT, Pham TP, Kyle D, Day NP, et al. Pharmacokinetics of oral artesunate in children with moderately severe Plasmodium falciparum malaria. Trans.R.Soc.Trop.Med.Hyg. 1997 Mar;91(2):195-8.

28. Newton P, Suputtamongkol Y, Teja-Isavadharm P, Pukrittayakamee S, Navaratnam V, Bates I, White N. Antimalarial bioavailability and disposition of artesunate in acute falciparum malaria. Antimicrob.Agents Chemother. 2000 Apr;44(4):972-7.

29. Nightingale CH. Pharmacokinetics and pharmacodynamics of newer macrolides. Pediatr.Infect.Dis.J. 1997 Apr;16(4):438-43.

30. Di Paolo A, Barbara C, Chella A, Angeletti CA, Del Tacca M. Pharmacokinetics of azithromycin in lung tissue, bronchial washing, and plasma in patients given multiple oral doses of 500 and 1000 mg daily. Pharmacol.Res. 2002 Dec;46(6):545-50.

31. Butcher GA, Sinden RE. Persistence of atovaquone in human sera following treatment: inhibition of Plasmodium falciparum development in vivo and in vitro. Am.J.Trop.Med.Hyg. 2003 Jan;68(1):111-4.

32. Taylor WR, White NJ. Antimalarial drug toxicity: a review. Drug Saf 2004;27(1):25-61.

33. Brewer TG, Peggins JO, Grate SJ, Petras JM, Levine BS, Weina PJ, Swearengen J, Heiffer MH, Schuster BG. Neurotoxicity in animals due to arteether and artemether. Trans.R.Soc.Trop.Med.Hyg. 1994 Jun;88 Suppl 1:S33-S36.

34. Wesche DL, DeCoster MA, Tortella FC, Brewer TG. Neurotoxicity of artemisinin analogs in vitro. Antimicrob.Agents Chemother. 1994 Aug;38(8):1813-9.

35. Kamchonwongpaisan S, McKeever P, Hossler P, Ziffer H, Meshnick SR. Artemisinin neurotoxicity: neuropathology in rats and mechanistic studies in vitro. Am.J.Trop.Med.Hyg. 1997 Jan;56(1):7-12.

36. Petras JM, Kyle DE, Gettayacamin M, Young GD, Bauman RA, Webster HK, Corcoran KD, Peggins JO, Vane MA, Brewer TG. Arteether: Risks of two-week administration in Macaca mulatta. American Journal of Tropical Medicine and Hygiene 1997 Apr;56(4):390-6.

37. Genovese RF, Newman DB, Li Q, Peggins JO, Brewer TG. Dose-dependent brainstem neuropathology following repeated arteether administration in rats. Brain Res.Bull. 1998;45(2):199-202.

38. Classen W, Altmann B, Gretener P, Souppart C, Skelton-Stroud P, Krinke G. Differential effects of orally versus parenterally administered qinghaosu derivative artemether in dogs. Exp.Toxicol.Pathol. 1999 Nov;51(6):507-16.

39. Genovese RF, Newman DB, Brewer TG. Behavioral and neural toxicity of the artemisinin antimalarial, arteether, but not artesunate and artelinate, in rats. Pharmacol.Biochem.Behav. 2000 Sep;67(1):37-44.

40. Nontprasert A, Pukrittayakamee S, Nosten-Bertrand M, Vanijanonta S, White NJ. Studies of the neurotoxicity of oral artemisinin derivatives in mice. Am.J.Trop.Med.Hyg. 2000 Mar;62(3):409-12.

41. Nontprasert A, Pukrittayakamee S, Dondorp AM, Clemens R, Looareesuwan S, White NJ. Neuropathologic toxicity of artemisinin derivatives in a mouse model. Am.J.Trop.Med.Hyg. 2002 Oct;67(4):423-9.

42. Nontprasert A, Pukrittayakamee S, Prakongpan S, Supanaranond W, Looareesuwan S, White NJ. Assessment of the neurotoxicity of oral dihydroartemisinin in mice. Trans.R.Soc.Trop.Med.Hyg. 2002 Jan;96(1):99-101.

43. Price R, van Vugt M, Phaipun L, Luxemburger C, Simpson J, McGready R, ter Kuile F, Kham A, Chongsuphajaisiddhi T, White NJ, et al. Adverse effects in patients with acute falciparum malaria treated with artemisinin derivatives. Am.J.Trop.Med.Hyg. 1999 Apr;60(4):547-55.

44. Adjuik M, Babiker A, Garner P, Olliaro P, Taylor W, White N. Artesunate combinations for treatment of malaria: meta-analysis. Lancet 2004 Jan 3;363(9402):9-17.

45. Bakshi R, Hermeling-Fritz I, Gathmann I, Alteri E. An integrated assessment of the clinical safety of artemether-lumefantrine: a new oral fixed-dose combination antimalarial drug. Trans.R.Soc.Trop.Med.Hyg. 2000 Jul;94(4):419-24.

46. van Vugt M, Angus BJ, Price RN, Mann C, Simpson JA, Poletto C, Htoo SE, Looareesuwan S, White NJ, Nosten F. A case-control auditory evaluation of patients treated with artemisinin derivatives for multidrug-resistant Plasmodium falciparum malaria. Am.J.Trop.Med.Hyg. 2000 Jan;62(1):65-9.

47. Kissinger E, Hien TT, Hung NT, Nam ND, Tuyen NL, Dinh BV, Mann C, Phu NH, Loc PP, Simpson JA, et al. Clinical and neurophysiological study of the effects of multiple doses of artemisinin on brain-stem function in Vietnamese patients. Am.J.Trop.Med.Hyg. 2000 Jul;63(1-2):48-55.

48. Hien TT, Turner GDH, Mai NTH, Phu NH, Bethell D, Blakemore WF, Cavanagh JB, Dayan A, Medana I, Weller RO, et al. Neuropathological assessment of artemether-treated severe malaria. Lancet 2003 Jul 26;362(9380):295-6.

49. Leonardi E, Gilvary G, White NJ, Nosten F. Severe allergic reactions to oral artesunate: a report of two cases. Transactions of the Royal Society of Tropical Medicine and Hygiene 2001;95(2):182-3.

50. Ruuskanen O. Safety and tolerability of azithromycin in pediatric infectious diseases: 2003 update. Pediatr.Infect.Dis.J. 2004 Feb;23(2 Suppl):S135-S139.

51. Looareesuwan S, Chulay JD, Canfield CJ, Hutchinson DB. Malarone (atovaquone and proguanil hydrochloride): a review of its clinical development for treatment of malaria. Malarone Clinical Trials Study Group. Am.J.Trop.Med.Hyg. 1999 Apr;60(4):533-41.

52. Cook JA, Randinitis EJ, Bramson CR, Wesche DL. Lack of a pharmacokinetic interaction between azithromycin and chloroquine. Am.J.Trop.Med.Hyg. 2006 Mar;74(3):407-12.

53. Gil VS, Ferreira MC, d'Alva FS, d'Abreu JA, Will IM, Gomes ML, Castelli F, Taylor WR, Olliaro P, D'Alessandro U. Efficacy of artesunate plus chloroquine for uncomplicated malaria in children in Sao Tome and Principe: a double-blind, randomized, controlled trial. Trans.R.Soc.Trop.Med.Hyg. 2003 Nov;97(6):703-6.

54. Crawley J. Reducing the burden of anemia in infants and young children in malaria-endemic countries of Africa: from evidence to action. Am J Trop Med Hyg 2004 Aug;71(2 Suppl):25-34.

55. Newbold C, Warn P, Black G, Berendt A, Craig A, Snow B, Msobo M, Peshu N, Marsh K. Receptor-specific adhesion and clinical disease in Plasmodium falciparum. Am.J.Trop.Med.Hyg. 1997 Oct;57(4):389-98.

56. Udomsangpetch R, Reinhardt PH, Schollaardt T, Elliott JF, Kubes P, Ho M. Promiscuity of clinical Plasmodium falciparum isolates for multiple adhesion molecules under flow conditions. J.Immunol. 1997 May 1;158(9):4358-64.

57. Heddini A, Pettersson F, Kai O, Shafi J, Obiero J, Chen Q, Barragan A, Wahlgren M, Marsh K. Fresh isolates from children with severe Plasmodium falciparum malaria bind to multiple receptors. Infect.Immun. 2001 Sep;69(9):5849-56.

58. Cattamanchi A, Kyabayinze D, Hubbard A, Rosenthal PJ, Dorsey G. Distinguishing recrudescence from reinfection in a longitudinal antimalarial drug efficacy study: comparison of results based on genotyping of msp-1, msp-2, and glurp. Am J Trop Med Hyg 2003 Feb;68(2):133-9.

59. Sulo J, Chimpeni P, Hatcher J, Kublin JG, Plowe CV, Molyneux ME, Marsh K, Taylor TE, Watkins WM, Winstanley PA. Chlorproguanil-dapsone versus sulfadoxine-pyrimethamine for sequential episodes of uncomplicated falciparum malaria in Kenya and Malawi: a randomised clinical trial. Lancet 2002 Oct 12;360(9340):1136-43.

60. Mung'ala-Odera V, Meehan R, Njuguna P, Mturi N, Alcock K, Carter JA, Newton CR. Validity and reliability of the 'Ten Questions' questionnaire for detecting moderate to severe neurological impairment in children aged 6-9 years in rural Kenya. Neuroepidemiology 2004 Jan;23(1-2):67-72.

61. Wijnhoven TM, de OM, Onyango AW, Wang T, Bjoerneboe GE, Bhandari N, Lartey A, al RB. Assessment of gross motor development in the WHO Multicentre Growth Reference Study. Food Nutr.Bull. 2004 Mar;25(1):S37-S45.

Appendix

Appendix I: Schedule of events

Schedule of events: Enrollment and each episode of malaria

S=Standard of care

R=Research activity

| Day of Study → | 0 | 1 | 2 | 3 | 7 | 14 | 21 | 28 | Unscheduled visit  (before d28) |
| --- | --- | --- | --- | --- | --- | --- | --- | --- | --- |
| Procedure ↓ |  |  |  |  |  |  |  |  |  |
| Informed consent (enrollment only) | R |  |  |  |  |  |  |  |  |
| History-comprehensive | S | R^a^ | R^a^ | R^a^ | R^a^ | R^a^ | R^a^ | R^a^ | S |
| History-possible adverse drug events and recent history |  | R | R | R | R | R | R | R | S |
| Questioning about antimalarial drug use | S | R | R | R | R | S | R | R | S |
| Physical examination | S | R | R | R | R | R | R | R | S |
| Neurological examination |  |  |  |  |  |  |  | R |  |
| Finger stick blood sample for malaria smear and hemoglobin. | S | R^a^ | R | R | R | R | R | R | S |
| Venous blood draw (parasite cryopreservation) | R |  |  |  | R^b^ | R ^b^ | R^b^ | R^b^ | R^b^ |
| CBC, ALT, creatinine | R |  |  |  |  | R |  |  |  |
| Finger stick blood sample for drug levels (r= at one of these time points only, research activity) | r^c^ |  | r^c^ | r | r | r | r | r | R^b^ |
| Assignment of study treatment group | R |  |  |  |  |  |  |  |  |
| Administration of drug | S | R | R |  |  |  |  |  |  |
| GPS mapping of house and household survey (enrollment only or if participant moves) | R |  |  |  |  |  |  |  |  |

^a^ As clinically indicated. Visits are part of research protocol but investigations of complaints are standard clinical care.

^b^ In cases of parasite density>10,000/mm^3^

^r^Participants randomly assigned to have blood drawn on two of these days

^c^Participants asked to return three hours after dose

Schedule of events: Routine visits and unscheduled visits (if malaria is diagnosed at any visit, follow schedule listed above)

|  | Routine visit 0-13^b^ | Unscheduled visit^c^ |
| --- | --- | --- |
| History-comprehensive | R | S |
| Questioning about medication use | R | S |
| Questioning about recent travel and bednet use | R |  |
| Physical examination | R | S |
| Malaria smear | R | S ^a^ |
| CBC, ALT, Creatinine, Neurological examination | Visits number 4, 8 and 13 |  |
| Additional evaluations | S ^a^ | S ^a^ |
| Counseling about next visit | R | R/S |

^a^ As clinically indicated

^b^Same procedure as premature termination visit

^c^If malaria is diagnosed, see schedule for malaria episode

Appendix II: Expected adverse reactions to study drugs

|  | Common adverse effects | Management of adverse effects | Rare adverse effects |
| --- | --- | --- | --- |
| CQ(32) | Pruritis  Nausea  Diarrhea  Dizziness | Repeat dose if vomiting occurs (total of 3 attempts) | None reported except for chronic administration (for weekly prophylaxis or treatment of rheumatoid disease) |
| AS(43) | Dizziness (15%)  Nausea (16%)  Vomiting (11%)  Diarrhea (34%)  Anorexia (1%)  Cannot distinguish these effects from underlying malaria disease | Repeat dose if vomiting occurs (total of 3 attempts) | Decreased reticulocyte count  Slight elevation of transaminase  Anaphylaxis |
| AP (Malarone package insert) | Vomiting (10%)  Pruritis (6%)  Diarrhea (6%)  Elevated transaminases (noted in Thai adults)-resolved spontaneously | Repeat dose if vomiting occurs (total of 3 attempts)  Follow transaminase level. Discontinue enrollment in study if abnormality does not resolve by next treatment episode. | Cutaneous reaction  Seizure and psychotic events (causal relationship not established) |
| AZ (Zithromax package insert) | Diarrhea (2.6%)  Abdominal pain (1.7%)  Vomiting (2.3%)  Nausea (0.4%)  Rash (0.6%) | Repeat dose if vomiting occurs.  Evaluate if rash occurs | Anemia  Leukopenia  Headache  Hyperkinesis  Dizziness  Agitation  Nervousness  Edema  Fever  Fungal superinfection  Cough  Urticaria  conjuctivitis |

Appendix III: Use of normal and abnormal values

Normal values for hematological and biochemical evaluations from healthy children in rural Mozambique (Quinto, et al. Tropical Medicine and International Health submitted, 2006) were used to define abnormalities in hemoglobin, ALT and creatinine.

ALT> 300IU/L is greater than 5 times the upper limit of normal

Creatinine>140 μmol/L is greater than 3 times the upper limit of normal.

|  | Grade 1 | Grade 2 | Grade 3 | Grade 4 |
| --- | --- | --- | --- | --- |
| **Hemoglobin for children less than or equal to 3 years of age** | 6.6-6.9 g/dL | 6.0-6.5 g/dL | <6.0 g/dL | Cardiac Failure secondary to anemia or requiring a transfusion |
| **Hemoglobin for children greater than 3 years of age** | 7.6-7.9 g/dL | 7.0-7.5 g/dL | <7.0 g/dL | Cardiac Failure secondary to anemia or requiring a transfusion |
| **ALT (IU/L)** | 75-150 | 151-300 | 301-600 | >600 |
| **Creatinine μmol/L** | 50-70 | 70-140 | 141-280 | >280 |

Other laboratory abnormalities will be graded according to the DMID Pediatric Toxicity Tables:

[http://icssc.org/documents/AE%20Manual%202003%20Appendices%20February_06_2003%20final.pdf](http://icssc.org/documents/AE Manual 2003 Appendices February_06_2003 final.pdf). Attached below

|  | Grade 1 | Grade 2 | Grade 3 | Grade 4 |
| --- | --- | --- | --- | --- |
| **Neutrophil count** | 750-1200/mm^3^ | 400-749/mm^3^ | 250-399/mm^3^ | <250/mm^3^ |


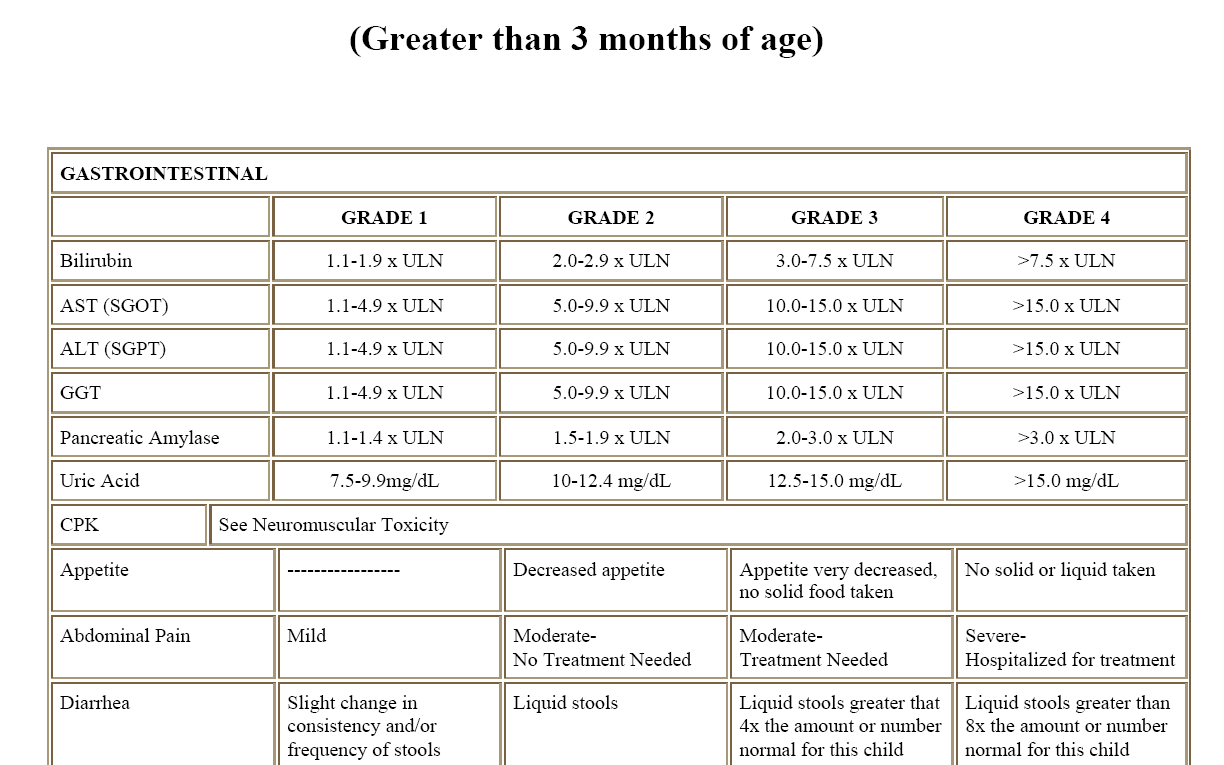

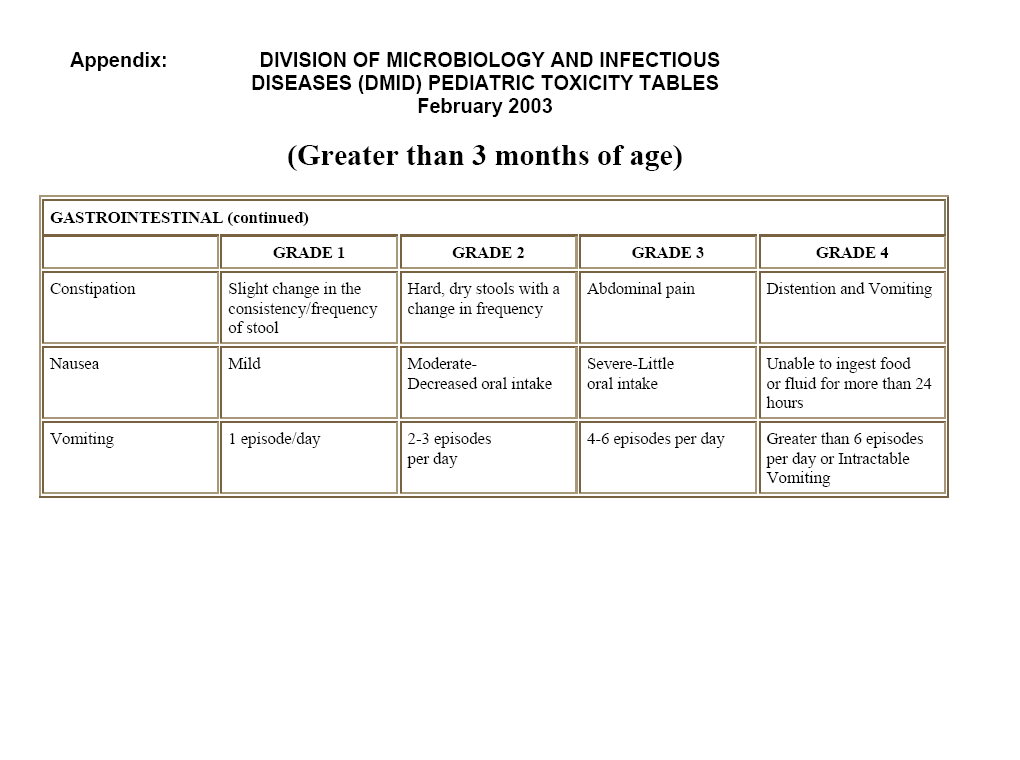


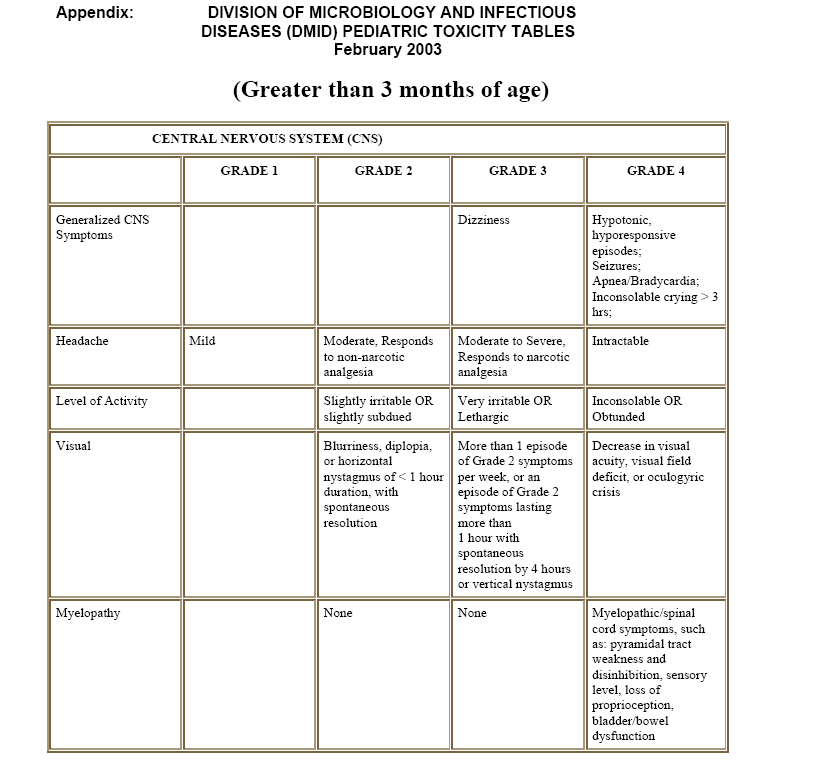


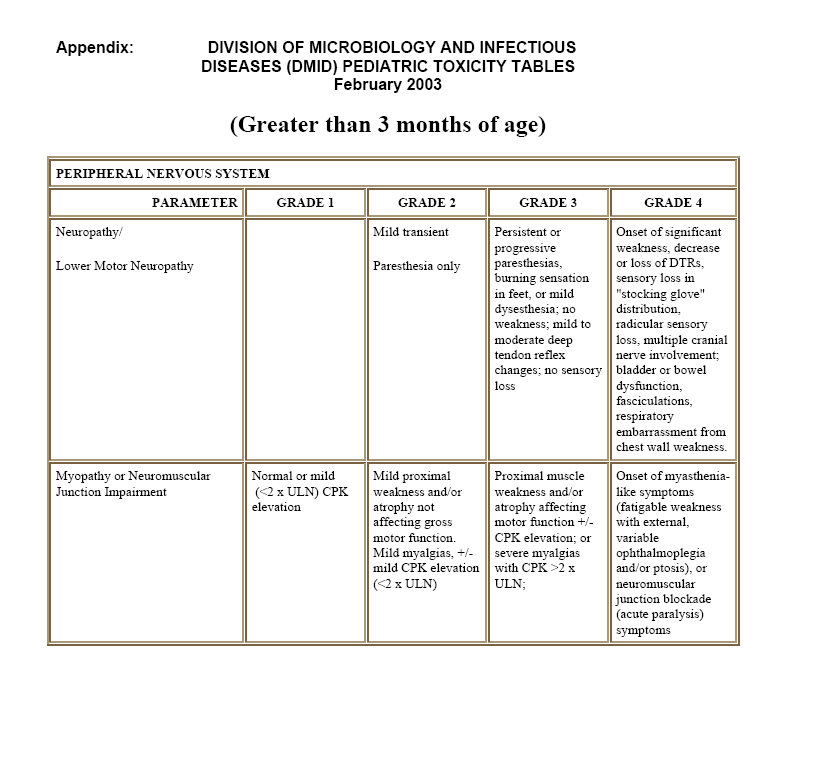


Appendix IV; Neurological Examination.

The following represents the minimum components of the scheduled neurological examination.

The neurological examination will include the following three parts:

- - 1. Screening for neurological impairment. Adapted from the ten question survey that has been validated for children in developing countries (60).

1. Compared with other children, does the child have any serious delay in sitting, standing or

walking?

2. Does the child have difficulty seeing, either in the daytime or at night?

3. Does the child appear to have difficulty hearing?

4. When you tell the child to do something, does he/she seem to understand what you are

saying? (Age 3+)

5. Does the child have difficulty in walking or moving his/her arms or does he/she have

weakness and/or stiffness in the arms or legs?

6. Does the child sometimes have fits, become rigid, or lose consciousness?

7. Does the child learn to do things like other children his/her age?

8. Does the child speak at all (can he/she make himself/herself understood in words; can

he/she say any recognizable words)? (After age 14 months)

9. For 3- to 9-year-old children ask: Is the child's speech in any way different from normal

(not clear enough to be understood by people other than his/her immediate family)?

For 2-year-old children ask: Can he/she name at least one object (for example, an animal, a

toy, a cup, a spoon)?

10. Compared with other children his/her age, does the child appear in any way backward,

dull or slow?

- - 1. Motor examination

From (61) with written instructions how to score these activities.

III Physical examination (minimum elements listed below)

- 1. Symmetry of movement and strength.
  2. Reflexes
  3. Age appropriate language based on the Denver Developmental Examination (observed by the clinician):
     - Has words 14-30 months
     - Had at least 5 words 18-30 months
     - Combines words OR names 2 objects/body parts 30-42 months
     - Speaks in understandable sentences 43+months

If the answer to any of the screening questions is yes, the participant will be referred to a pediatrician for a complete evaluation. If the participant falls outside the standard error bar above or there is an abnormality in the physical examination, the evaluation will be repeated within 28 days. If the abnormality persists, the participant will be referred to a pediatrician for a complete evaluation.
